# Further contributions to the staphylinid fauna of New Brunswick, Canada, and the USA, with descriptions of two new *Proteinus* species (Coleoptera, Staphylinidae)

**DOI:** 10.3897/zookeys.573.7830

**Published:** 2016-03-24

**Authors:** Reginald P. Webster, Anthony E. Davies, Jan Klimaszewski, Caroline Bourdon

**Affiliations:** 1 24 Mill Stream Drive, Charters Settlement, NB, Canada E3C 1X1; 2 Agriculture and Agri-Food Canada, Canadian National Collection of Insects, Arachnids and Nematodes, Ottawa, Ontario, K1A 0C6, Canada; 3 Natural Resources Canada, Canadian Forest Service - Laurentian Forestry Centre, 1055 du P.E.P.S., P.O. Box 10380, Stn. Sainte-Foy, Québec, Quebec, Canada G1V 4C7

**Keywords:** Staphylinidae, *Proteinus*, new records, New Brunswick, Canada, USA

## Abstract

This paper treats the discovery of new species and new records of Staphylinidae from the subfamilies Omaliinae, Proteininae, Tachyporinae, Oxytelinae, Scydmaeninae, Steninae, Euaesthetinae, Pseudopsinae, Paederinae, and Staphylininae for the province of New Brunswick and other provinces of Canada, and the USA. We report here two species new to science, three new North American records, nine new Canadian records, two new USA records, and 50 new provincial records. The following are the species new to science: *Proteinus hughesi* Webster & Davies, **sp. n.** and *Proteinus sweeneyi* Webster & Klimaszewski, **sp. n.** (Proteininae). *Sepedophilus immaculatus* (Stephens) and *Carpelimus erichsoni* (Sharp), *Carpelimus mundus* (Sharp) are newly recorded from North America. New Canadian records are as follows: *Carpelimus difficilis* (Casey), *Carpelimus gracilis* (Mannerheim), *Carpelimus lacustris* (Notman), *Carpelimus probus* (Casey), *Carpelimus pusillus* (Gravenhorst), *Carpelimus rivularis* (Motschulsky), *Carpelimus spretus* (Casey), *Carpelimus weissi* (Notman) (Oxytelinae), and *Edaphus lederi* Eppelsheim (Euaesthetinae). This is the first record of the genus *Edaphus* for Canada. *Bledius basalis* LeConte and *Carpelimus obesus* (Kiesenwetter) (Oxytelinae) are removed from the faunal list of New Brunswick. *Proteinus acadiensis* Klimaszewski and *Proteinus pseudothomasi* Klimaszewski are newly recorded from the USA and several provinces of Canada. Habitat data from New Brunswick are provided for most of the species treated in this contribution.

## Introduction

In recent years, the Staphylinidae of New Brunswick have been studied intensively. In a series of papers published in a Special Issue of ZooKeys (186), Biosystematics and Ecology of Canadian Staphylinidae, 184 species of Staphylinidae were newly reported from New Brunswick in the following 15 subfamilies: Omaliinae, Micropeplinae, Phloeocharinae, Olisthaerinae, Habrocerinae (Webster et al. 2012d), Pselaphinae (Webster et al. 2012a), Tachyporinae (Webster et al. 2012e), Aleocharinae (Webster et al. 2012b), Scaphidiinae, Piestinae, Osorinae, Oxytelinae (Webster et al. 2012f), Oxyporinae (Webster and DeMerchant 2012a), Paederinae (Webster and DeMerchant 2012b), and Staphylininae (Webster et al. 2012c). Later, Klimaszewski et al. (2013, 2014, 2015a, b) added 19 species in the genera *Atheta*, *Clusiota*, *Dinaraea*, *Gnathusa*, *Mniusa*, *Ocyusa*, and *Mocyta* to the faunal list of New Brunswick as a result of new species descriptions and new records. In a recent review of the *Euaesthetus* (Euaesthetinae) of North America, Puthz (2014) added nine members of this genus to the faunal list of the province. In this special issue, Webster et al. (2016b) and Klimaszewski et al. (2016) report another 66 Aleocharinae new to New Brunswick, including 30 species new to science, one new North American record, six new Canadian records, and 29 new provincial records.

During the last several years new provincial and Canadian records from the subfamilies Omaliinae, Proteininae, Tachyporinae, Oxytelinae, Scydmaeninae, Steninae, Euaesthetinae, Pseudopsinae, Paederinae, and Staphylininae have been documented from the province of New Brunswick. New jurisdictional data from other provinces of Canada and the USA for some of the species treated in this publication were found in material in the Canadian National Collection, Ottawa. The purpose of this paper is to report on these new discoveries.

## Methods and conventions

**Collection methods.** Various methods were employed to collect the specimens reported in this study. Details are outlined in Webster et al. (2009, Appendix). Some specimens were collected from Lindgren funnel trap samples during a study to develop improved tools and methods for detection of invasive species of Cerambycidae. These traps are visually similar to tree trunks and are often effective for sampling species of Coleoptera that live in microhabitats associated with standing trees (Lindgren 1983). Traps were baited with various combinations of lures for detecting Cerambycidae. See Webster et al. (2012b), Hughes et al. (2014), and Webster et al. (2016a) for details of the lures and methods used to deploy Lindgren traps and collect samples. A description of the habitat was recorded for all specimens collected during this survey. Locality and habitat data are presented as on the labels for each record. Information is separated by a // in the data presented from each specimen where more than one label is present. Habitat information is summarized in the natural history section for each species.

**Specimen preparation and photography.** Many specimens were dissected to confirm their identity. The genital structures were dehydrated in absolute alcohol and mounted in Canada balsam on celluloid microslides and then pinned with the specimen from which they originated. Images of the entire body and the genital structures were taken using an image processing system (Nikon SMZ 1500 stereoscopic microscope; Nikon Digit-like Camera DXM 1200F, and Adobe Photoshop software).

**Distribution.** All species are cited with current Distribution in Canada and Alaska, using abbreviations for the state, provinces, and territories. New provincial records are indicated in **bold** under **Distribution in Canada and Alaska**. The following abbreviations are used in the text:

AB Alberta


AK Alaska


BC British Columbia


MB Manitoba


NB New Brunswick


NF & LB Newfoundland and Labrador*


NS Nova Scotia


NT Northwest Territories


NU Nunavut


ON Ontario


PE Prince Edward Island


QC Quebec


SK Saskatchewan


YT Yukon Territory


*Newfoundland and Labrador are each treated separately under the current Distribution in Canada and Alaska.

USA state abbreviations follow those of the US Postal Service. Acronyms of collections referred to in this study where specimens reside are as follows:

AFC
 Natural Resources Canada, Canadian Forest Service - Atlantic Forestry Centre, Fredericton, New Brunswick, Canada 


CNC
Canadian National Collection of Insects, Arachnids, and Nematodes, Agriculture and Agri-Food Canada, Ottawa, Ontario, Canada 


NBM
 New Brunswick Museum, Saint John, New Brunswick, Canada 


HNHM
Hungarian Natural History Museum, Budapest, Hungary 


RWC
 Reginald Webster Collection, Charters Settlement, New Brunswick, Canada 


## Results and discussion

### Species accounts

Species with a † are adventive to Canada, species with a ‡ are either Holarctic or adventive to Canada, species with a * are Holarctic. The determination that a species was a new record is based on information in the print version of Bousquet et al. (2013). The classification used below follows Bouchard et al. (2011).

### Family Staphylinidae Latreille, 1806

#### Subfamily Omaliinae MacLeay, 1825

The Omaliinae occurring in NB were reviewed by Webster et al. (2012d). They newly recorded 11 species for the province. Here, we report two more species.

##### Tribe Anthophagini C.G. Thomson, 1859

###### Olophrum boreale

Taxon classificationAnimaliaColeopteraStaphylinidae

(Paykull, 1792)*

####### Material examined.

**New Brunswick, Restigouche Co.**, Kedgwick Rd. at Fog Brook, 47.8367°N, 67.8739°W, 21.VI.2011, R.P. Webster & M. Turgeon // *Carex* marsh with brook, treading emergent *Carex* into water (1 sex undetermined, RWC); Summit Lake, 47.7825°N, 68.3199°W, 7.VI.2011, R.P. Webster // Lake margin, *Carex* marsh, treading *Carex* hummocks and emergent vegetation (1 sex undetermined, RWC). **Ontario**, Moosonee, 51.24690°N, 80.68102°W [at sewage lagoon] Rep. 3 mesic, yellow pan 23-26.VI.2010, NBP field party, M3MY331 (1, CNC).

####### Distribution in Canada and Alaska.

AK, YT, NT, BC, AB, SK, MB, **ON**, QC, **NB** (Bousquet et al. 2013). This species is newly recorded from ON and NB.

####### Natural history.

Specimens were collected during June by treading emergent *Carex* into water in a *Carex* marsh along a lake margin and in a *Carex* marsh near a small stream. The specimen from ON was captured in a yellow pan trap near a sewage lagoon in June. Campbell (1983) reported this species from similar habitats elsewhere in its range.

##### Tribe Omaliini MacLeay, 1825

###### Phyllodrepa humerosa

Taxon classificationAnimaliaColeopteraStaphylinidae

(Fauvel, 1878)

####### Material examined.

**New Brunswick, Sunbury Co.**, Sunpoke Lake, 45.7656°N, 66.5550°W, 18.VI-9.VII.2012, 9-20.VII.2012, C. Alderson & V. Webster // Red oak forest near seasonally flooded marsh, Lindgren funnel trap in canopy of *Quercus rubra* (1 ♂, RWC); **Sunbury Co.**, Gilbert Island, 45.8770°N, 66.2954°W, 28.V-12.VI.2012, C. Alderson, C. Hughes, & V. Webster // Hardwood forest, Lindgren funnel trap in canopy of *Juglans cinerea* (1 ♂, RWC). **York Co.**, Charters Settlement, 45.8395°N, 66.7391°W, 4.V.2004, 16.IV.2005, R.P. Webster, coll. // Mixed forest, in decaying (moldy) corncobs & cornhusks (1 ♂, 1 ♀, RWC); same locality data and collector but 19.VI.2005 // Mixed forest, in dung trap (1 ♀, RWC); 15 km W of Tracy off Rt. 645, 45.6848°N, 66.8821°W, 20.VI-6.VII.2011, M. Roy & V. Webster // Old red pine forest, Lindgren funnel trap (1 ♂, RWC).

####### Distribution in Canada and Alaska.

AB, ON, QC, **NB**, NS (Bousquet et al. 2013).

####### Natural history.

This species was collected in a red oak (*Quercus rubra* L.) forest, a hardwood forest on an island, a mixed forest, and a red pine (*Pinus resinosa* Ait.) forest. Specimens were captured in Lindgren funnel traps in most of the above forest types, others were found in decaying (moldy) corncobs and cornhusks, and one was collected in a dung (pitfall) trap. Adults were collected in April, May, June, and July.

#### Subfamily Proteininae Erichson, 1839

The Proteininae are a relatively small subfamily of Staphylinidae with two genera of small, relatively broad-bodied species in North America (Newton et al. 2000). Both genera, *Megarthrus* and *Proteinus*, occur in NB. Members of the two genera are found in decaying fungi, carrion, and plant debris and are probably saprophagus or mycophagus (Newton et al. 2000). Four species (two from each genus) have been recorded from NB (Bousquet et al. 2013). The two *Proteinus* species known from NB, *Proteinus acadiensis* Klimaszewski and *Proteinus pseudothomasi* Klimaszewski, were described from specimens collected at the Acadia Research Forest (Klimaszewski et al. 2005). Since that publication, three additional species of *Proteinus* have been discovered in NB, one being a new provincial record, the other two are new species and are described below. New jurisdictional data from NB, several Canadian provinces, and the USA are reported for *Proteinus acadiensis* Klimaszewski and *Proteinus pseudothomasi* Klimaszewski.

##### Tribe Proteinini Erichson, 1839

###### Proteinus acadiensis

Taxon classificationAnimaliaColeopteraStaphylinidae

Klimaszewski, 2005

####### Comments.

Originally described from NB, and later reported from a yellow birch (*Betula alleghaniensis* Britt.) forest in QC by Klimaszewski et al. (2007), an examination of RWC and CNC material revealed additional specimens from NB, as well as from across Canada and the northeastern United States.

####### Material examined.

**Canada, Alberta**, Twp 25, Rge 3, W 5 Mer, 13.VII.1998, B.F. & J.L. Carr, Lot 1, pitfall traps baited with dead shrew (1 ♂, CNC); Twp 26, Rge 5, W 5 Mer, 10.VII.1980, B.F. & J.L. Carr, Lot 1, baited pitfall traps north end of slough (2 ♂, CNC); Twp 28, Rge 5, w 5 Mer, 3.VIII.1979, B.F. & J.L. Carr, Lot 3 (1 ♂, CNC); Twp 86, Rge 3, W 6 Mer, 29.VII.1989, B.F. & J.L. Carr, Lot 3, large beaver pond below dam on Montagneuse Lake outlet (1 ♂, CNC); Waterton Lakes National Park, 22.VII.1980, D.E. Bright, pantrap (1 ♂, CNC). **British Columbia**, Peachland, 17.VIII.1919, J.B. Wallis (1 ♂, 2 ♀, CNC); Yoho National Park, Amiskwi River [near junction of Kiwetinok River], 6000’, 7.VIII.1971, J.M. & B.A. Campbell (1 ♂, 1 ♀, CNC). **Manitoba**, 1 km north of Onanole, 29.VIII.1979, S.J. Miller, berlese ex mushrooms, aspen woods (1 ♂, CNC); Riding Mountain National Park [RMNP], 6 km E of Clear Lake, 24.VIII.1979, D.B. Lyons, ex agaric mushrooms (7 ♂, CNC); RMNP, Jet Trail, 21.VIII.1979, D.B. Lyons, ex agaric mushroom (1 ♂, CNC); RMNP, Katherine Lake, 13.VI.1979, D.B. Lyons, ex *Russula* sp. (5 ♂, CNC); RMNP, Moon Lake, 21.VIII.1979, S.J. Miller, berlese ex mushrooms (6 ♂, CNC); same data except berlese litter under *Acer negundo* (1 ♂, CNC); RMNP, near refuse pit, 16.VIII.1979, S.J. Miller, berlese ex moose dung (7 ♂, CNC). **New Brunswick**, **Albert Co.**, Caledonia Gorge P.N.A. [Protected Natural Area], 45.7941°N, 64.7736°W, 13.IX.2011, R.P. Webster // near Crooked Creek, mixed forest (red spruce & yellow birch), in decaying gilled mushrooms (1 ♂, RWC). [**Kent Co.**], Kouchibouguac Nat. Park [KNP], 29.VIII.1977, G.A. Calderwood, 5902Z (2 sex undetermined, CNC); same data but 5944P (1 sex undetermined, CNC); KNP, 17.IX.1977, J.M. Campbell, 5976V (48 sex undetermined, CNC); same data but 5975U (1 sex undetermined, CNC); KNP, 21.IX.1977, Campbell & Smetana, 6014H (9 ♂, CNC); same data but S.J. Miller, 6018L (1 sex undetermined, CNC). **Queens Co.**, Cranberry Lake P.N.A., 46.1125°N, 65.6075°W, 22.IX.2009, R.P. Webster, coll. // Red oak forest, in decaying gilled mushrooms (7 ♂, RWC). **Restigouche Co.** Gounamitz Rd., near Gounamitz R., 47.6102°N, 67.7902°W, 15.X.2013, R.P. Webster // Old spruce & balsam fir forest, in rotting *Tricholoma* sp. (1 ♂, RWC). **Sunbury Co.**, Acadia Research Forest, 45.9799°N, 66.3394°W, 18.IX.2007, R.P. Webster, coll. // Road 7 Control, mature red spruce & red maple forest, in gilled mushroom (1 ♂, RWC). **Nova Scotia**, Cape Breton Highlands Nat. Park [CBHNP], Aspy River trail, 90 m, PG795856, 23.IX.1984, J.M. Campbell & A. Davies, , ex mushrooms on tree stumps (1 ♀, CNC); CBHNP, Lone Shieling, PG729861, 75 m, 19.IX.1984, J.M. Campbell & A. Davies, ex mushrooms (2 ♀, CNC); CBHNP, Pleasant Bay, 25 m, PG682872, 14.IX.1984, J.M. Campbell & A. Davies, sifting litter & moss (13 sex undetermined, CNC); same data but 21.IX.1984, sifting alder litter (3 sex undetermined, CNC); same data but ex mushrooms (21 sex undetermined, CNC). **Ontario**, Constance Bay, X.1970, S. Peck (1 ♂, 4 ♀, CNC); 7 km S Westport, Chaffeys Locks Biol. Station, 44°34'08N, 76°19'15W, 23.X.1985, A. Davies, alder-birch litter at edge of marsh (2 ♂, CNC); same data except ex mushrooms in birch litter (4 ♂, CNC); same data except 26.IX.1986, J.M. Campbell & A. Davies, ex *Boletus* mushrooms (2 ♂, 8 ♀, CNC); same data except 3.X.1986, A. Davies & L. Dumouchel, ex mushrooms (1 ♂, 4 ♀, CNC). **Quebec**, Gatineau Park, Pinks Lake, 45.4684'N 75.8117'W, A. Davies, 24.IX.1982, berlese mushrooms (1 ♂, 2 ♀, CNC). **USA, Maine**, **Kennebec Co.**, Belgrade, 44.474°N, 69.835°W, 17.XII.1983, R.E. Nelson, mixed duff and moss (1 ♂, CNC). **New Hampshire, Carroll Co.**, 6 mi NW Bartlett, Nancy Brook Trail, 1500’, 9.IX.1987, A. Davies & Y. Bousquet, ex *Russula* mushrooms (6 ♂, 4 ♀, CNC); same data except A. Davies, sifting litter and frass in tree holes (1 ♂, CNC). **Coos Co.**, 2 mi NE Crawford Depot, 2100’, 13.IX.1987, J.M. Campbell & A. Davies, ex mass of mushrooms on large stump (2 ♂, 2 ♀, CNC); 17 km S Gorham, Glen Ellis Falls, 1900’, 8.IX.1987, J.M. Campbell & A. Davies, sifting litter by stream (9 ♂, 8 ♀, CNC).

####### Distribution in Canada and Alaska.

**BC**, **AB**, **MB**, **ON**, QC, NB, **NS** (Klimaszewski et al. 2005, Klimaszewski et al. 2007). In Canada, *Proteinus acadiensis* is newly recorded from BC, AB, ON, and NS and is reported for the first time for the USA from ME and NH. This species is transcontinental in Canada.

####### Natural history.

In NB, specimens were collected from decaying gilled mushrooms and rotting *Tricholoma* sp. mushroom in a red oak forest, in mixed forests, and an old spruce (*Picea*) & balsam fir (*Abies balsmea* (L.) Mill.) forest. Elsewhere, specimens were found in *Boletus* mushrooms, agaric mushrooms, *Russula* sp. mushrooms, a mass of mushrooms on a large stump, from berlese samples from mushrooms, moose dung, and leaf litter, sifted from mushrooms, a pitfall trap baited with dead shrew, sifted from various kinds of litter, such as mixed duff and moss, alder (*Alnus*) litter, and litter and frass in tree holes. Adults were collected during July, August, September, October, and December with most records from August and September. Little was previously known about the habitat associations of this species.

###### Proteinus hughesi

Taxon classificationAnimaliaColeopteraStaphylinidae

Webster & Davies sp. n.

http://zoobank.org/609F629A-7B0B-4665-B50F-A1578F05A24E

Figs 1–4

####### Holotype (male).

**Canada, New Brunswick, Northumberland Co.**, ca. 2.5 km W of Sevogle, 47.0879°N, 65.8585°W, 1.X.2013, R.P. Webster // Old *Pinus banksiana* forest, in rotting gilled mushroom (CNC). **Paratypes: New Brunswick, Madawaska Co.**, near Falls Brook Falls, 47.5877°N, 68.3687°W, 16.X.2013, R.P. Webster & M. Turgeon // Spruce & balsam fir forest, in decaying mushroom (1 ♂, RWC); 47.5877°N, 68.3687°W, 16.X.2013, R.P. Webster // Mature hardwood forest, in decaying *Tricholoma* sp. (3 ♂, RWC). **Restigouche Co.**, 1.5 km S. of Quebec [border], 425 m elev., 47.9058°N, 68.1505°W, 22.VI.2010, R.P. Webster // Boreal forest, small cold-shaded brook, splashing gravel on gravel bar (1 ♂, RWC); Gounamitz Rd. near Gounamitz R., 47.6102°N, 67.7902°W, 15.X.2013, R.P. Webster // Old spruce & balsam fir forest in rotting *Tricholoma* sp. (1 ♀, 2 ♂, RWC). **York Co.**, Charters Settlement, 45.8395°N, 66.7391°W, 18.X.2007, R.P. Webster // Mixed forest, in decaying (moldy) corncobs & cornhusks (2 ♂, RWC); same data as previous but 15.IV.2004 // mixed forest, in compost (decaying vegetable matter) (1 ♀, RWC). **Newfoundland**, Little Grand Lake, Bakeapple Brook, 15.VII-25.VIII.1992, old fir, pitfall (1 ♂, 2 ♀, CNC); Corner Brook, Cooks Pond - lower 40y fir, 23-30.VII.1992, pitfall (1 ♀, CNC). **Nova Scotia**, Cape Breton Highlands Nat. Park [CBHNP], Lone Shieling, 100 m, PG729861, 3-5.VI.1983, H. Goulet, pans, malaise (2 ♂, 2 ♀, CNC); same locality, 6-7.VI.1983, H. Goulet, forest malaise (1 ♂, CNC); same locality, 9-10.VI.1983, H. Goulet, forest malaise (1 ♂, CNC); same locality, 19.VI.1983, Y. Bousquet, interception (1 ♂, 2 ♀, CNC); same locality, 25.VI.1983, Y. Bousquet, pans (26 ♂, CNC); same locality, 28.VI.1983, R. Vockeroth, pans, malaise (2 ♂, CNC); same locality, 1.VII.1983, R. Vockeroth, malaise trough (1 ♂, CNC); CBHNP, MacKenzie Mtn., 300 m, PG648868, 11-13.VI.1983, H. Goulet, birch fern pans (1 ♂, CNC); same locality, 22.VI.1983, Y. Bousquet, pans (1 ♂, CNC). **Quebec**, Old Chelsea, 6.X.1956, J.R. Vockeroth, on *Hygrophorus puniceus* Fr. (1 ♂, 2 ♀, CNC); **Co. Vaudreuil**, Rigaud end Ch. de la Croix, 5.V.1988, 950, A. and Z. Smetana (1 ♂, CNC). **USA: Kentucky, Edmonson Co.**, Mammoth Cave Nat. Park, Running Branch Cave, 5.V.1972, S. Peck, Ber 235 (1 ♂, CNC).

####### Etymology.

This species is named in honor of Cory Hughes (AFC), who worked with us on many of the projects that provided the new records for this paper and many previous publications. Without his assistance, many of these records would not have been possible.

####### Description.

**Figures 1–4.**
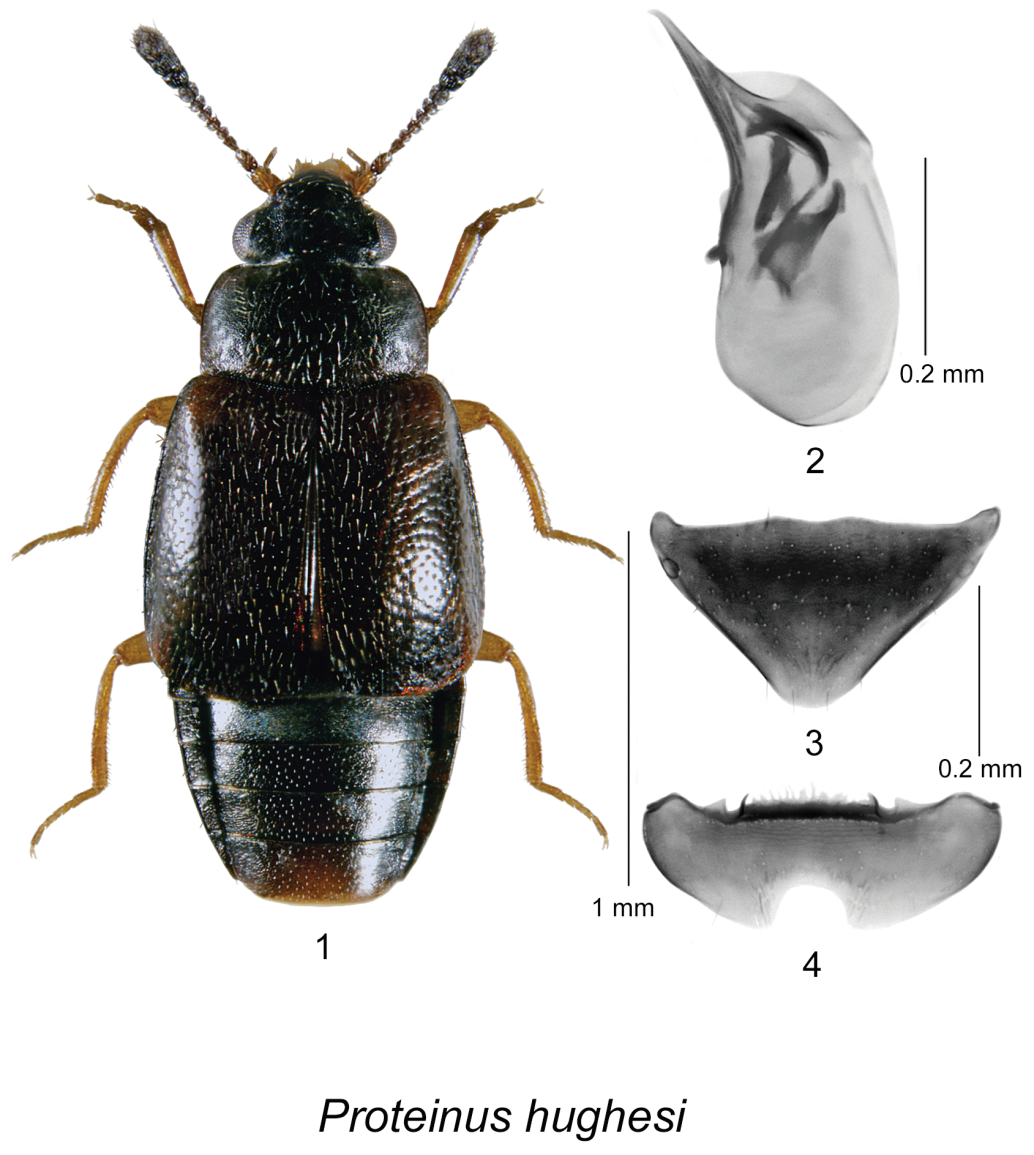
*Proteinus hughesi* Webster & Klimaszewski, sp. n.: **1** male habitus in dorsal view **2** median lobe of aedeagus in lateral view **3** male tergite VII **4** male sternite VII.

Body length 2.0–2.2 mm, head black, pronotum dark piceous brown and lighter than head; elytra piceous brown, often slightly lighter than pronotum, first two antennal segments testaceous, second segment sometimes darker, remaining segments dark brown becoming slightly darker towards last segment; legs testaceous; forebody and elytra with pubescence sparse, recumbent, directed posteriad; head and pronotum with distinct isodiametric microsculpture throughout, stronger on head, punctures widely spaced, shallow; elytra with punctation coarse, sparse, with little microsculpture, thus appearing glossy; pronotum with lateral margin arcuate in anterior third, then nearly straight to hind margin, hind angle nearly rectangular, narrowly rounded, hind margin sinuate; mesosternum with disc transversely rugose, with anteromedial carinae long, divergent, well-separated; mesosternal process very narrow, spiniform between middle coxae, without carina or pubescence; metasternum distinctly finely scalloped along anterior marginal bead, process very broadly rounded between middle coxae, disc sparsely pubescent; body shape and proportions as in Fig. 1. **Male.** First segment of front tarsus expanded, remaining segments normal; posterior margin of middle trochanter almost straight, with row of 3–6 short peg setae; middle tibia distinctly arcuate with a series of peg-like setae along apical 2/3 of inner margin; hind trochanter with single peg seta at middle of posterior margin; metasternum with broad glabrous impunctate area in front of hind coxae. Tergite VII triangular in shape, posterior margin rounded at apex (Fig. 3); posterior margin of sternite VII broadly rounded with a deep semicircular emargination (Fig. 4). Median lobe of aedeagus without angular subapical part in lateral view, with dark internal structures as illustrated (Fig. 2). **Female.** Similar to male but first tarsal segment only slightly expanded; middle tibia nearly straight, inner margin lacking peg-like setae. Tergite VII similar in shape to that of male; sternite VII without emargination.

####### Comments.

We compared the genitalia of the types of all known North American species and available illustrations of the genitalia of all Palearctic species and found none matching this species which led to the conclusion that this species was undescribed.

####### Distribution.

This species is recorded in Canada from QC, NB, NF, and NS, and in the USA, from KY.

####### Natural history.

In NB, this species was found in spruce and balsam fir forests, an old jack pine (*Pinus banksiana* Lamb.) forest, a mixed forest, and in a “Boreal” forest (spruce and fir). Most adults were found in rotting *Tricholoma* and other decaying gilled mushrooms. One individual was collected from gravel on a gravel bar along a small shaded brook, two were found among decaying (moldy) corncobs and cornhusks, and one from compost. Adults were collected in April, June, and October. Elsewhere, specimens were collected from malaise traps, pan traps, interception traps, and pitfall traps during May and June.

###### Proteinus parvulus

Taxon classificationAnimaliaColeopteraStaphylinidae

LeConte, 1863

Figs 5–9

####### Material examined.

**Alberta**, Canmore, 5.VIII.1961, B.F. & J.L. Carr, Lot 3, toadstools in pine + spruce forest, (1 ♂, CNC); Twp 28, Rge 5, W 5 Mer, 10.IX.1981, B.F. & J.L. Carr, Lot 1, fungus & litter in pine, spruce, poplar forest (1 ♂, CNC); same data except 3.VIII.1979, Lot 3, Twp 34, Rge 7, W 5 Mer, 9.VIII.1980, B.F. & J.L. Carr, Lot 1, evergreen logs (1 ♂, CNC). **British Columbia**, Yoho National Park, Amiskwi River [near junction of Kiwetinok River], 6000’, 7.VIII.1971, J.M. & B.A. Campbell (5 ♂, 4 ♀, CNC). **Manitoba**, Riding Mountain National Park [RMNP], 6 km E of Clear Lake, 24.VIII.1979, D.B. Lyons, ex agaric mushrooms (18 ♂, 1 ♀, CNC); same data except ex pile of rotting mushrooms (2 ♂, CNC); RMNP, Katherine Lake, 13.VI.1979, D.B. Lyons, ex *Russula* sp. (2 ♂, CNC); RMNP, Moon Lake, 21.VIII.1979, S.J. Miller, berlese ex mushrooms (3 ♂, 1 ♀, CNC). **New Brunswick, Madawaska Co.**, near Falls Brook Falls, 47.5877°N, 68.3687°W, 16.X.2013, R.P. Webster & M. Turgeon // Spruce & balsam fir forest, in decaying mushroom (1 ♂, RWC); **Saint John Co.**, Dipper Harbour, 45.1176°N, 66.3806°W, 24.VIII.2006, R.P. Webster, coll. // Red spruce & balsam fir forest, in decaying gilled mushrooms (5 ♂, RWC); same data and forest type but 12.IX.2006, on gilled mushrooms (4 ♀, 1 ♂, RWC). **Saskatchewan**, Hwy. 955, 63 km N La Loche, Clearwater River crossing campground, 4.VIII.1984, B.F. & J.L. Carr, Lot 1, almost dry pine/aspen litter (1 ♂, CNC). **Yukon Territory**, Dawson City, 11.VII.1968, J.M. Campbell & A. Smetana, sifting old *Boletus* mushrooms (1 ♂, 1 ♀, CNC); same data except 16.VII.1968, sifted rotten mushrooms (42 ♂, 38 ♀ CNC); Mile 14 W of Dawson, 3000’, 3.VIII.1949, P. Bruggemann (2 ♂, CNC); Marsh Lake, 5.VIII.1987, B.F. & J.L. Carr, Lot 1, in fungus (1 ♂, 2 ♀, CNC).

####### Diagnosis.

**Figures 5–9.**
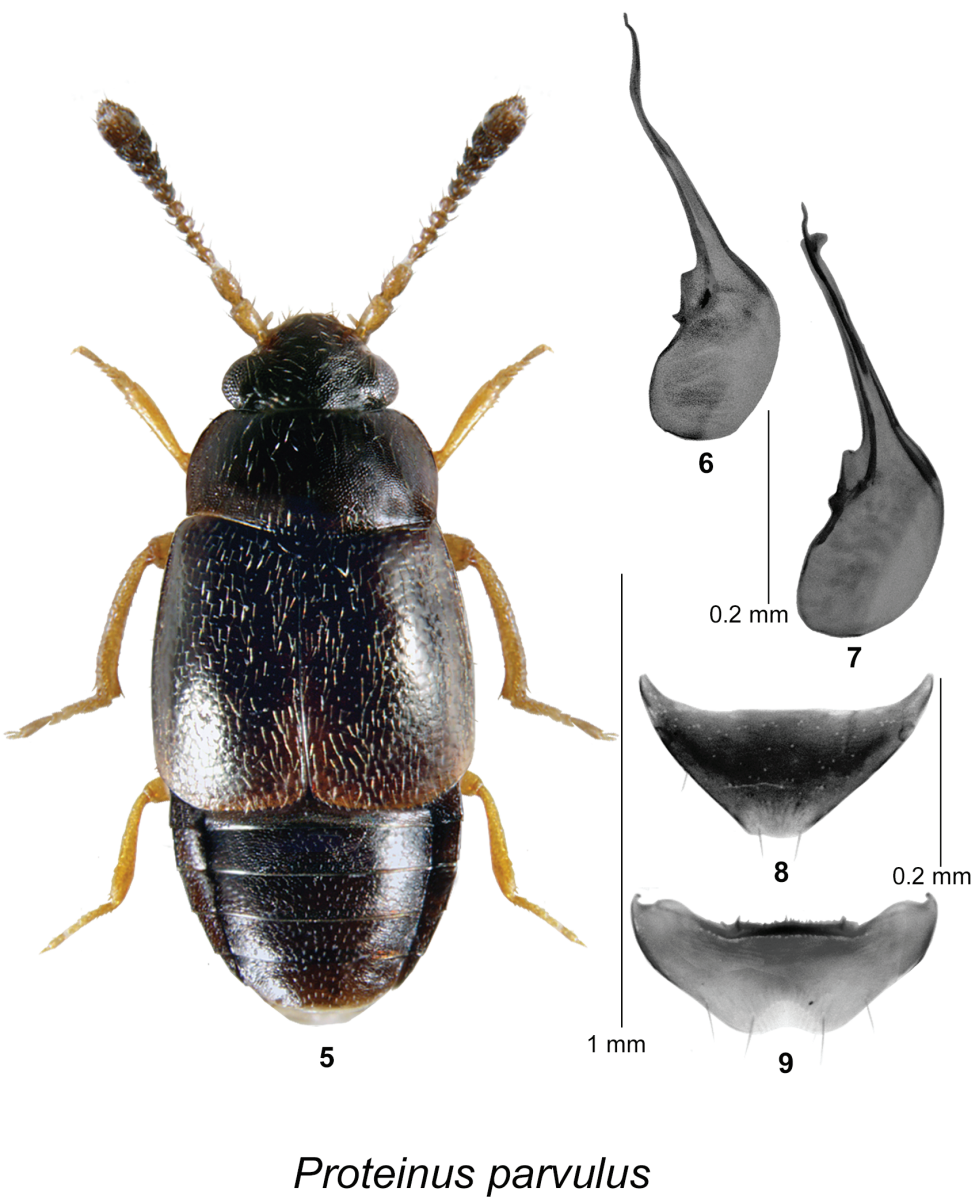
*Proteinus parvulus* LeConte: **5** male habitus in dorsal view **6–7** median lobe of aedeagus in lateral view **8** male tergite VII **9** male sternite VII.

Body length 1.5–1.8 mm, head black, pronotum and elytra dark brown and lighter than head; first two antennal segments testaceous, remaining segments dark brown becoming slightly darker toward last segment; legs testaceous; forebody and elytra with pubescence sparse, recumbent, directed posteriad; head and pronotum with distinct isodiametric microsculpture throughout, slightly stronger on head, punctures widely spaced, shallow; elytra with punctation coarse, sparse, with little microsculpture, thus appearing glossy; lateral margin of pronotum broadly arcuate, widest at middle, hind angle obtuse, slightly rounded; hind margin sinuate; mesosternum with disk irregularly rugulose, with anteromedial carinae short, subparallel, well-separated, mesosternal process narrow, with fine, short carina between middle coxae, gradually tapering to acute apex; metasternum depressed along anterior marginal bead, process very broadly rounded between middle coxae, disk sparsely pubescent; body shape and proportions as in Fig. 5. **Male.** Front tarsus with first tarsomere expanded, twice as long as wide, remaining tarsomeres normal; middle trochanter with posterior margin evenly rounded, without peg setae; middle femur with posterior margin broadly expanded in apical half, with series of 2–4 stout bullet-shaped setae along expansion and one closer to base; middle tibia very broadly arcuate, with small fin-like projection at apex of inner margin, without a series of peg-like setae; hind trochanter densely punctulate; hind tibia with inner margin abruptly narrowing in apical 1/4 in ventral aspect, with sparse short erect setae. Tergite VII triangular in shape, posterior margin truncate at apex (Fig. 8); posterior margin of sternite VII broadly rounded with a shallow semicircular emargination (Fig. 9). Median lobe of aedeagus with angular subapical part in lateral view, with indistinct internal structures as illustrated (Figs 6, 7). **Female.** Similar to male but first tarsal segment only slightly expanded; middle tibia nearly straight. Tergite VII similar in shape to that of male; sternite VII without emargination.

####### Distribution in Canada and Alaska.

**YK, BC**, **AB**, **SK**, **MB**, ON, **NB** (Bousquet et al. 2013). *Proteinus parvulus* was described from “Lake Superior” but was reported from localities in ON from that region (Batchewana Bay and Michipicoten River) soon thereafter by Hubbard and Schwarz (1878), which probably represents the material on which LeConte based his description. This species is newly recorded from YK, BC, AB, SK, MB, and NB in Canada. This species is transcontinental in Canada.

####### Natural history.

In NB, *Proteinus parvulus* was found in a spruce and fir forest and a red spruce forest. Most specimens were found in decaying gilled mushrooms. Adults were collected in August, September, and October. Elsewhere in Canada, adults were collected during July and August from fungus and litter in a pine, spruce, and poplar (*Populus*) forest, in toadstools in a pine and spruce forest, in agaric mushrooms, in a pile of rotting mushrooms, in *Russula* sp. mushrooms, sifted from old *Boletus* mushrooms and rotting mushrooms, and from mushrooms.

###### Proteinus pseudothomasi

Taxon classificationAnimaliaColeopteraStaphylinidae

Klimaszewski, 2005

####### Comments.

Originally described from NB, and later reported from a yellow birch forest in QC by Klimaszewski et al. (2007), an examination of CNC material revealed additional specimens of this rare species from across Canada, as well as the eastern United States. Additional locality data from NB in the RWC are also included.

####### Material examined.

**Canada, Alberta**, Twp 107, Rge 16, W 5 Mer, trail into forest just outside Machesis campground entrance, 25.VII.1989, B.F. & J.L. Carr, in fungus (1 ♂, CNC); Waterton Lakes National Park, Chief Mtn Hwy Km 9, 4500’, 17.VII.1980, H.J. Teskey (1 ♂, CNC). **New Brunswick**, **Carleton Co.**, Wakefield, Meduxnekeag Valley Nature Preserve, 46.1927°N, 67.6803°W, 16.IX.2006, R.P. Webster, coll. // **Queens Co.**, Cranberry Lake P.N.A., 46.1125°N, 65.6075°W, 22.IX.2009, R.P. Webster, coll. // Red oak forest, in decaying gilled mushrooms (6 ♂, RWC). **Saint John Co.**, Dipper Harbour, 45.1176°N, 66.3806°W, 24.VIII.2006, R.P. Webster, coll. // Red spruce & balsam fir forest, in decaying gilled mushrooms (1 ♂, RWC). **York Co.**, Canterbury, trail to “Browns Mtn. Fen”, 45.8954°N, 67.6307°W, 7.IX.2007, R.P. Webster, coll. // Mixed forest, in decaying gilled mushrooms (2 ♀, RWC). **Newfoundland**, Corner Brook, Loggers Sch. Rd. – U 60y fir, 25.VI-24.VII.1992, pitfall, (1 ♂, CNC). **Ontario**, 7 km S Westport, Chaffeys Locks Biol. Station, 44°34'08N, 76°19'15W, 23.X.1985, A. Davies, birch + maple litter beside logs (2 ♂, CNC). **Quebec**, Parc de la Gatineau, Lac Bourgeois, 7.VII.1982, J.E.H. Martin (1 ♂, CNC); same data except 12.VII.1982 (1 ♂, CNC); Parc de la Gatineau, 2 km S Lac Mousseau, 26.V-2.VI.1980, E. Rickey & A. Davies, flight intercept trap at beaver pond (1 ♂, CNC); Parc de la Gatineau, Pinks Lake, 45.4684°N 75.8117°W, A. Davies, 4.IX.1982, berlese mushrooms (1 ♂, CNC). **U.S.A., Illinois**, **Union Co**., Pine Hills Field Station, 15-22.V.1967, J.M. Campbell (1 ♂, 7 ♀, CNC). **Kentucky**, Pennyroyal State Park near Dawson Springs, 22.III.1983, J.M. Campbell (2 ♂, CNC). **Pennsylvania**, **Fulton Co.**, Cowan Gap State Park, 26-28.V.1981, S. Peck, oak forest UV (1 ♂, 2 ♀, CNC).

####### Distribution in Canada and Alaska.

**AB**, **ON**, QC, NB, **NF** (Klimaszewski et al. 2005, Klimaszewski et al. 2007, Bousquet et al. 2013). In Canada, this species is newly recorded from AB, ON, and NF, and is reported for the first time for the USA, based on records from IL, KY, and PA.

####### Natural history.

In NB, *Proteinus pseudothomasi* was found in a red spruce and balsam fir forest, a mixed forest, and a red oak forest during August and September. All specimens were found in decaying gilled mushrooms. Elsewhere, specimens were found in fungus, collected from Berlese sample from mushrooms, a flight intercept trap, from birch and maple litter beside logs, and at UV light in an oak forest. Adults were collected from April to October. Little was previously known about the habitat associations of this species.

###### Proteinus sweeneyi

Taxon classificationAnimaliaColeopteraStaphylinidae

Webster & Klimaszewski sp. n.

http://zoobank.org/BD6DAAE6-5C65-42B9-99CD-011785F11EAB

Figs 10–13

####### Holotype (male).

**Canada, New Brunswick, Saint John Co.**, Dipper Harbour, 45.1169°N, 66.3771°W, 7.V.2006, R.P. Webster // Sea beach, in decaying sea wrack on gravel and sand // PHOTO 2015-007, C. Bourdon (CNC). **Paratypes: Manitoba**, 1 km north of Onanole, 29.VIII.1979, S.J. Miller, berlese ex mushrooms, aspen woods (2 ♂, CNC); Riding Mountain National Park, near refuse pit, 16.VIII.1979, S.J. Miller, berlese ex moose dung (1 ♀, CNC); same data except 15.VIII.1979, ex mammal burrows (2 ♂, CNC). **New Brunswick, Madawaska Co.**, 47.5984°N, 68.3667°W, 16.X.2013, R.P. Webster // Mature hardwood forest, in decaying *Tricholoma* sp. (1 ♀, RWC). **Queens Co.**, Cranberry Lake P.N.A., 45.1125°N, 65.6075°W, 24.IV-5.V.2009, R. Webster & M.-A. Giguère // red oak forest, Lindgren funnel trap (1 ♂, RWC). **Restigouche Co.**, Jacquet River Gorge P.N.A., 47.8207°N, 65.9955°W, 15.VI.2009, R.P. Webster // Black spruce forest with *Populus* sp., in gilled mushroom (1 ♂, RWC). **Saint John Co.**, same data as holotype (1 ♀ RWC); same data and collector as previous but 15.V.2006 // Upper margin of sea beach, in decaying sea wrack under alders (♀, 1 ♂, RWC). **York Co.**, New Maryland, Charters Settlement, 45.8395°N, 66.7391°W, 29.III.2006, R.P. Webster, coll. // Mixed forest, flight intercept trap adjacent to composter (2 ♂, RWC). **Nova Scotia**, Cape Breton Highlands Nat. Park, Lone Shieling, PG729861, 100 m, 6-7.VI.1983, H. Goulet, forest malaise (1 ♂, CNC); same data except 9.VI.1983 (1 ♂, CNC); same data except 11-13.VI.1983 (1 ♂, CNC); same data except VII.1983, R. Vockeroth, malaise trap (1 ♂, 1 ♀, CNC); same data except 19.VI.1983, Y. Bousquet, interception (1 ♂, 1 ♀, CNC). **Ontario**, 7 km S Westport, Chaffeys Locks Biol. Station, 44°34'08N, 76 °19'15W, 23.X.1985, A. Davies, birch + maple litter beside logs (1 ♂, 1 ♀, CNC); **Quebec**, Parc de la Gatineau, Blind Lake, 8.V.1988, A. & Z. Smetana (1 ♂, 1 ♀, CNC); Parc de la Gatineau, visitor centre, 45.5068'N, 75.8161'W, 15-22.IV.1987, J. Denis, J. Huber & A. Davies, emergence trap at woodpile (7 ♂, 7 ♀, CNC); same data except 21-28.IV.1987 (2 ♂, 4 ♀, CNC).

####### Etymology.

This species is named in honor of Jon Sweeney (AFC). His long-term project on the development of a general attractant for the detection of invasive species of Cerambycidae provided numerous new species records from NB for the Cerambycidae and many other Coleoptera families.

####### Description.

**Figures 10–13.**
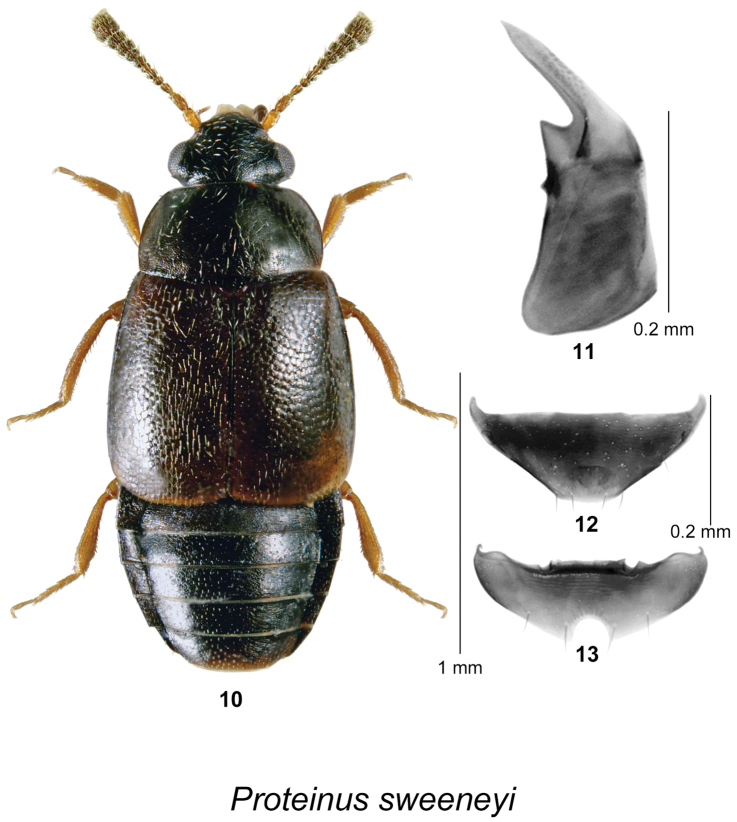
*Proteinus sweeneyi* Webster & Klimaszewski, sp. n.: **10** male habitus in dorsal view **11** median lobe of aedeagus in lateral view **12** male tergite VII **13** male sternite VII.

Body length 1.7–2.0 mm, head black, pronotum dark brown and lighter than head; elytra brown to dark brown, lighter than pronotum; first two antennal segments testaceous, remaining segments dark brown becoming darker toward last segment; legs testaceous; forebody and elytra with pubescence sparse, recumbent, directed posteriad; pronotum with microsculpture distinct, dilated on sides and at base, becoming isodiametric near center, punctures widely spaced, shallow; elytra with punctation coarse, sparse, with little microsculpture, thus appearing glossy; pronotum with lateral margin arcuate in anterior two-thirds, then almost straight to hind margin, widest just before hind angle, hind angle obtuse, narrowly rounded, hind margin sinuate; mesosternum with disk irregularly rugulose, with anteromedial carinae forming semi-circular ridge with anteromedial margin, mesosternal process broad, gradually tapering to narrowly rounded apex, with long, very fine median carina; metasternum very broadly rounded between middle coxae, disk sparsely, coarsely pubescent; body shape and proportions as in Fig. 10. **Male.** Front tarsus with first tarsomere expanded, parallel-sided, 3x as long as wide, as long as next 4 together, remaining segments normal; posterior margin of middle trochanter almost evenly rounded, without peg setae; middle femur with hind margin expanded in apical half, with 2-3 coarse setae on expansion; middle tibia broadly arcuate, inner margin without peg-like setae or projection; hind trochanter explanate, with dense patch of short pile covering half of posteroventral surface; hind tibia expanded in ventral aspect, widest at distal third, inner margin obliquely excavate in apical half, with dense patch of short erect setae near apex. Tergite VII triangular in shape, posterior margin truncate at apex (Fig. 12); posterior margin of sternite VII broadly rounded with a deep semicircular emargination (Fig. 13). Median lobe of aedeagus with an angular subapical part in lateral view, without obvious darkened internal structures, other characters as illustrated (Fig. 11). **Female.** Similar to male, but first tarsal segment only slightly expanded; middle tibia nearly straight. Tergite VII similar in shape to that of male; sternite VII without emargination.

####### Distribution.

This species is known from MB, ON, QC, NB, and NS in Canada.

####### Natural history.

In NB, this species was found in a red oak forest, mature hardwood forest, black spruce (*Picea mariana* (Mill.) BSP) forest with *Populus* sp., a mixed forest, and on a sea beach. Specimens were collected from decaying *Tricholoma* sp., a gilled mushroom, decaying sea wrack, a Lindgren funnel trap, and a flight intercept trap adjacent to a composter. Elsewhere, this species was collected from mushrooms, moose dung, a mammal burrow, birch (*Betula*) and maple (*Acer*) litter beside logs, and from an emergence trap at a wood pile; some specimens were captured in malaise and flight intercept traps. Adults were collected from March to October.

####### Comments.

We compared the genitalia of the types of all known North American species and available illustrations of the genitalia of all Palearctic species and found none matching this species, which led to the conclusion that this species was undescribed. There are several other species of *Proteinus* (*Proteinus atomarius* Erichson, *Proteinus basalis* Mäklin, *Proteinus brachypterus* (Fabricius), *Proteinus collaris* Hatch, *Proteinus densipennis* Bernhauer, *Proteinus limbatus* Mäklin [all examined]) reported from Canada, including a number of undescribed species (in CNC), that are mostly western in distribution. However, it is beyond the scope of this publication to present a comparison of our newly described species with all of the other North American species until this genus is revised. We therefore provide comparisons only for the five species known to occur in NB. The external morphology has a limited number of diagnostic features and the shape and structure of the median lobe of the aedeagus are the most reliable for species level identification.

*Proteinus hughesi*, *Proteinus parvulus*, and *Proteinus sweeneyi* are very similar in coloration and general habitus but differ most notably in characters of the pronotum, middle tibia, and the shape of the aedeagus (Figs 2, 6–7, 11) in the males. Males of *Proteinus hughesi* have a row of peg-like setae along the inner margin of the mesotibia, which are absent in *Proteinus parvulus* and *Proteinus sweeneyi* (Fig. 1). The mesotibia of *Proteinus parvulus* bears a small fin-like projection at the apex of the inner margin (Fig. 5), while in *Proteinus sweeneyi* the middle tibia is without any modification of the inner margin (Fig. 10). Females are more difficult to separate but they differ in the shape and microsculpture of the pronotum. In *Proteinus sweeneyi*, the pronotal microsculpture is dilated laterally and basally, becoming isodiametric only near the center; in *Proteinus parvulus* and *Proteinus hughesi*, the microsculpture is isodiametric on nearly all of the pronotum. In *Proteinus hughesi*, the pronotum is widest near the base, with the lateral margins arcuate on the anterior third, then straight to the almost rectangular hind angles; in *Proteinus parvulus*, the lateral margin is arcuate throughout, with the widest point near the middle and the hind angle is obtuse. *Proteinus acadiensis* and *Proteinus pseudothomasi* differ from the above three species by their coloration (light brown or reddish brown), the lack of modification of the middle and hind legs in the males (the middle tibia is arcuate in *Proteinus acadiensis*), and the shape of the genitalia (see Figs 4, 31, 32 for *Proteinus pseudothomasi* and Figs 5, 33 for *Proteinus acadiensis* in Klimaszewski et al. (2005)).

#### Subfamily Tachyporinae MacLeay, 1825

The Tachyporinae occurring in NB were reviewed by Webster et al. (2012e). They recorded 23 species for the province for the first time. Here, we report three additional species.

##### Tribe Mycetoporini C.G. Thomson, 1859

###### Mycetoporus rufohumeralis

Taxon classificationAnimaliaColeopteraStaphylinidae

Campbell, 1991

####### Material examined.

**New Brunswick, Queens Co.**, Grand Lake Meadows P.N.A., 45.8227°N, 66.1209°W, 5-17.VIII.2011, M. Roy & V. Webster // Old silver maple forest & seasonally flooded marsh, Lindgren funnel trap (1, RWC).

####### Distribution in Canada and Alaska.

AK, YT, BC, AB, ON, **NB**, NS (Bousquet et al. 2013).

####### Natural history.

One individual was captured in a Lindgren funnel trap in an old silver maple (*Acer saccharinum* L.) forest. Campbell (1991) reported this species from river debris and moss and leaf litter.

##### Tribe Tachyporini MacLeay, 1825

###### Sepedophilus immaculatus

Taxon classificationAnimaliaColeopteraStaphylinidae

(Stephens, 1832)†

Figs 14–15

####### Material examined.

**New Brunswick, York Co.** 15 km W of Tracy, off Rt. 645, 45.6848°N, 66.8821°W, 16-30.VI.2010, R. Webster & C. MacKay, coll. // Old red pine forest, Lindgren funnel trap (1 ♂, RWC).

####### Distribution in Canada and Alaska.

**NB (New North American record)**. This is the first record of this Palaearctic species for North America. *Sepedophilus immaculatus* is a common species in Europe, occurring in southeastern, eastern, middle Europe, the southern part of northern Europe to western Siberia, Great Britain south to Algeria, Tunisia, Cyprus, Iran, Lebanon, and Turkey (Schülke & Smetana 2015: 472).

####### Natural history.

In Europe, *Sepedophilus immaculatus* occurs in various forest types and habitats, including beech (*Fagus*) forest, steppe with shrubbery, mixed hardwood forest, mixed forest, *Quercus* forest, flood plain forest, *Quercus*–*Carpinus* [oak–hornbeam] forest, and stream ravine (Schülke 2011). Specimens were found in leaf litter, a pinewood wood pile, flood debris, in refuse, vinegar trap, chestnut bark, and sifted from litter in a *Platanus* tree hole in the above habitats.

####### Comments.

**Figures 14–17.**
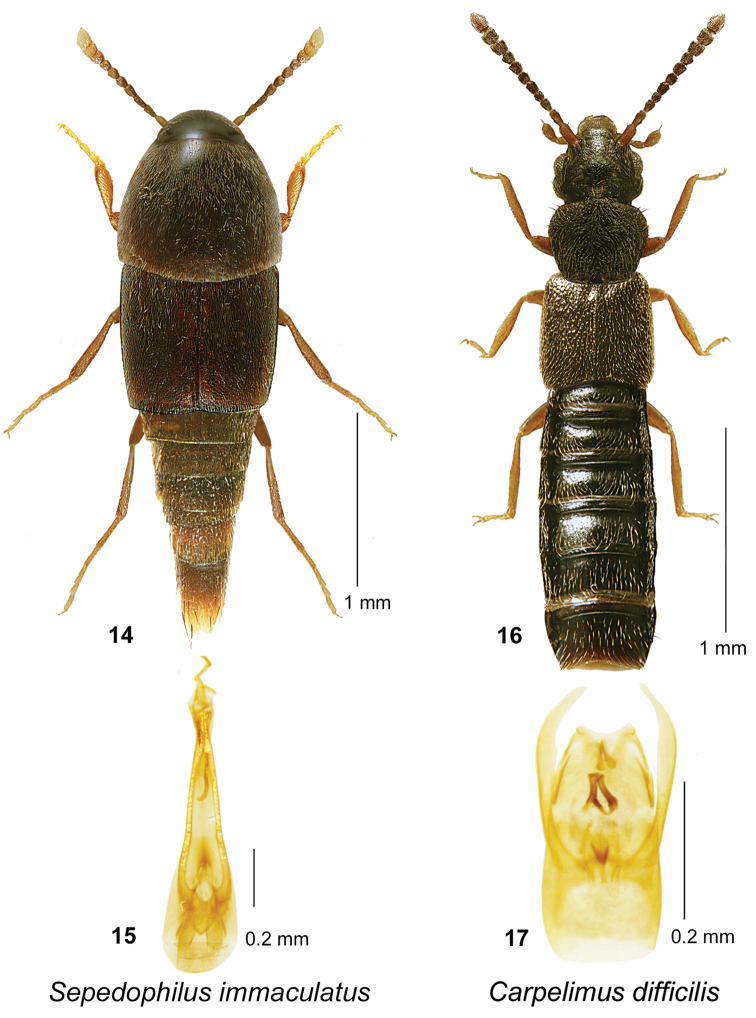
*Sepedophilus immaculatus* (Stephens): **14** habitus in dorsal view **15** aedeagus in ventral view. *Carpelimus difficilis* (Casey): **16** habitus in dorsal view **17** aedeagus in ventral view.

This adventive species is distinguished from other North American species of *Sepedophilus* by the distinctively shaped internal structures of the aedeagus (Figs 14–15) (See Schülke 2011 for additional details; Figs 3 & 4, p 1613), with a “corkscrew-shaped” sclerite in the internal sac, and the complete lack of lateral bristles on the abdomen.

###### Tachinus (Tachinus) elongatus

Taxon classificationAnimaliaColeopteraStaphylinidae

Gyllenhal, 1810*

####### Material examined.

**New Brunswick, Northumberland Co.**, ca. 2.5 km W of Sevogle, 47.0879°N, 65.8585°W, 25.VI-9.VII.2014, C. Alderson & V. Webster // Old *Pinus banksiana* forest, Lindgren funnel trap (1, RWC).

####### Distribution in Canada and Alaska.

AK, YT, NT, BC, AB, SK, MB, ON, QC, **NB**, LB, NF (Bousquet et al. 2013).

####### Natural history.

One individual was captured in a Lindgren funnel trap in an old jack pine forest. Elsewhere, specimens have been collected from under stones in damp areas, on banks of small streams, in wet moss, from under damp decayed leaves and rubbish, and occasionally in dung and carrion (Campbell 1973).

#### Subfamily Oxytelinae Fleming, 1821

Webster et al. (2012f) newly recorded six species of Oxytelinae in their review of NB species of this subfamily. Makranczy (2014), in his revision of the *Ochthephilus*, described *Ochthephilus ashei* Makanczy, based in part on a specimen from NB, and reported *Ochthephilus forticornis* (Hochhuth and *Ochthephilus planus* (LeConte) from the province, both of which were new provincial records. Here, we report an additional 15 species for the province, including eight species that are new for Canada and two species new to North America.

##### Tribe Blediini Ádám, 2001

###### Bledius basalis

Taxon classificationAnimaliaColeopteraStaphylinidae

LeConte, 1863

####### Note.

*Bledius basalis* was reported by Majka et al. (2008) from Jemseg, NB from moist bare clay in a silver maple forest 70 km inland from the Bay of Fundy. Majka considered this location unusual as this species is typically associated with slightly vegetated sand flats adjacent to the ocean (Herman 1976). This specimen, determined by C. Majka, was re-examined and was found to be *Bledius annularis* LeConte, a species previously known from the province. In NB, *Bledius annularis* typically occurs on moist clay banks along shaded brooks and rivers (Webster, unpublished). *Bledius basalis* is accordingly removed from the faunal list of the province.

###### Bledius opaculus

Taxon classificationAnimaliaColeopteraStaphylinidae

LeConte, 1863

####### Material examined.

**New Brunswick, Kent Co.**, Kouchibouguac N.P., South Kouchibouguac Dune, 46.8251°N, 64.9079°W, 19.VII.2014, R.P. Webster // Sand/clay intertidal area behind sand dune, splashing sand/clay (2 ♂, 1 ♀, 8 sex undetermined, RWC; 4 sex undetermined, NBM).

####### Distribution in Canada and Alaska.

QC, **NB**, NS, PE, NF (Bousquet et al. 2013).

####### Natural history.

Specimens were collected by splashing sand/clay in an intertidal area with sparse vegetation behind a barrier sea beach (sand dune).

##### Tribe Oxytelinae Fleming, 1821

###### Carpelimus

Taxon classificationAnimaliaColeopteraStaphylinidae

Leach, 1819

####### Note.

Examples of each of the *Carpelimus* species reported below were determined by György Makranczy, Hungarian Natural History Museum, Hungary. These specimens are currently in the Hungarian Natural History Museum. Other *Carpelimus* specimens of the species reported below were determined by R.P. Webster, based on the above determinations. The aedeagi of the adventive species were also compared to the illustrations provided by Gildenkov (2015) for additional confirmation. György Makranczy is currently working on a much-needed revision of the North American members of this genus, for which all published records (see below) must be re-examined, as the last revision was by Casey (1889). Eight of the 12 species reported below are new records for Canada. However, until a revision of the Nearctic fauna is completed, we have no idea what the true distributions might be in the rest of the continent, aside from the types and the records reported here. There are at least another 10 species of *Carpelimus* from NB that cannot be named at this time.

###### Carpelimus difficilis

Taxon classificationAnimaliaColeopteraStaphylinidae

(Casey, 1889)

Figs 16–17

####### Material examined.

**Canada, New Brunswick, Albert Co.**, Caledonia Gorge P.N.A., 45.8380°N, 64.8484°W, 3.VII.2011, R.P. Webster // near Turtle Creek, old-growth hardwood forest, mossy seepage with some *Carex*, sifting saturated moss (1 ♂, RWC). **Carleton Co.**, Wakefield [Belleville], Meduxnekeag Valley Nature Preserve, 46.1931°N, 67.6825°W, 14.IX.2005, R.P. Webster // River margin, in flood debris (1 ♀, RWC); Richmond, Hovey Hill Protected [Natural] Area, 46.1157°N, 67.7624°W, 14.V.2006, R.P. Webster // Mixed forest, margin of vernal pond in moist leaves (1 ♀, RWC). **Queens Co.**, near Queenstown, 45.6904°N, 66.1455°W, 13.V.2008, R.P. Webster, coll. // Old-growth hardwood forest, in leaf litter near seepage & brook (1 ♂, HNHM). **Sunbury Co.**, Maugerville, Portobello Creek N.W.A., 45.8882°N, 66.4248°W, 5.VII.2005, R.P. Webster // Silver maple swamp, muddy river bank (1 ♂, RWC). **York Co.**, Charters Settlement, 45.8331°N, 66.7410°W, 16.IV.2004, R.P. Webster // Mature red spruce & cedar forest, in moss & litter near brook (1 sex undetermined, RWC); Kingsclear, Mazerolle Settlement, 45.8717°N, 66.8273°W, 28.IV.2006, R.P. Webster // Cedar forest, in leaves on muddy soil near brook (1 ♂, HNHM; 1 ♂, RWC); Rt. 645 at Beaver Brook, 45.6840°N, 66.8679°W, 3.V.2008, R.P. Webster // Red maple/alder swamp, in moist leaves near small vernal pools near small stream (1 ♀, HNHM; 1 ♂, RWC).

####### Distribution in Canada and Alaska.

**NB (New Canadian record).** The type series was collected in NC and MD in the USA. *Carpelimus difficilis* was later reported by Ulke (1902) from the District of Columbia (DC) and by Notman (1920) from NY State, but these records need to be confirmed.

####### Natural history.

In NB, adults of *Carpelimus difficilis* were usually associated with the margins of streams and vernal ponds in various forest types. Specimens were found in saturated moss in a seepage near a creek in an old-growth hardwood forest, in leaf litter near a seepage and brook in an old-growth hardwood forest, in moss and litter near a brook in a mature red spruce and eastern white cedar (*Thuja occidentalis* L.) forest, among leaves on muddy soil near a brook in a cedar forest, in flood debris on a river margin, in moist leaves on a vernal pond margin in a mixed forest, and a red maple (*Acer rubrum* L.)/alder swamp, and on a muddy river bank. Adults were collected during April, May, July, and September.

###### Carpelimus erichsoni

Taxon classificationAnimaliaColeopteraStaphylinidae

(Sharp, 1871)†

Figs 18–19

####### Material examined.

**Canada, New Brunswick, York Co.**, Charters Settlement, 45.8395°N, 66.7391°W, 26.IX.2008, 19.V.2011, R.P. Webster // Mixed forest, in decaying (moldy) corncobs & cornhusks (1 ♂, HNHM; 3 ♂, RWC).

####### Distribution in Canada and Alaska.

**NB (New North American record).** Schülke and Smetana (2015: 786) list this species from much of southern Europe from Russia (Southern Territory) and Yugoslavia south to Algeria and east to the Netherlands and Belgium. Callot (2013) recently cited a record from France, but this was not accepted/seen by Schülke. This species is adventive to NB, possibly via the Mediterranean region, although this is not the typical source of adventive species in the region.

####### Natural history.

All NB specimens were collected in May and September from decaying (moldy) corncobs and cornhusks, near a plastic composter.

####### Comments.

*Carpelimus erichsoni* is very similar externally to *Carpelimus bilineatus* Stephens (also an adventive species) but has differently shaped internal structures of the aedeagus (Makranczy 2002, Gildenkov 2015).

**Figures 18–21.**
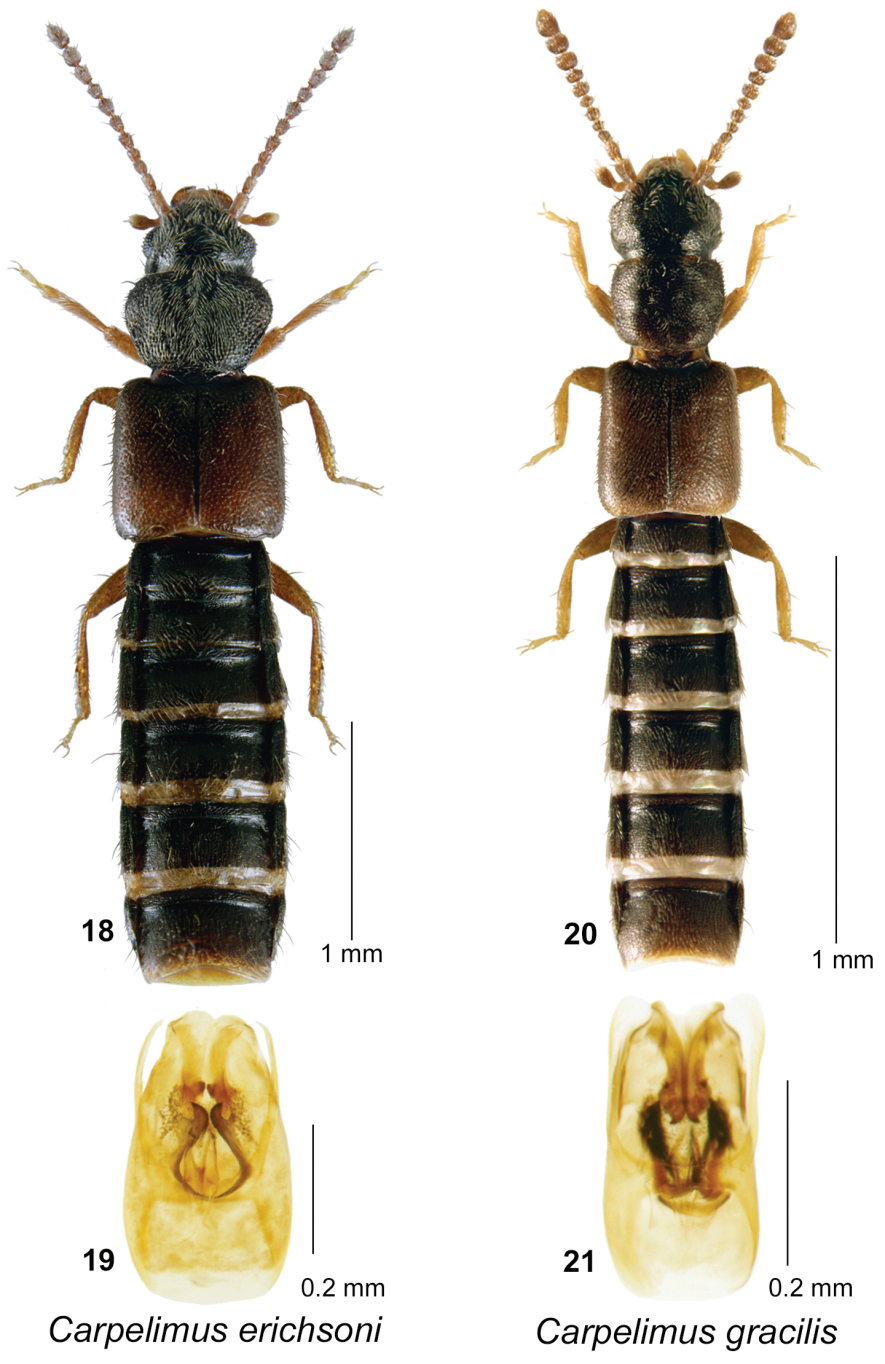
*Carpelimus erichsoni* (Sharp): **18** habitus in dorsal view **19** aedeagus in ventral view. *Carpelimus gracilis* (Mannerheim): **20** habitus in dorsal view **21** aedeagus in ventral view.

###### Carpelimus gracilis

Taxon classificationAnimaliaColeopteraStaphylinidae

(Mannerheim, 1831)†

Figs 20–21

####### Material examined.

**Canada, New Brunswick, Restigouche Co.**, Wild Goose Lake, 420 m elev., 47.8540°N, 68.3219°W, 7.VI.2011, R.P. Webster & M. Turgeon// Lake margin with emergent *Carex* and grasses, treading *Carex* and grasses (1 ♀, RWC). **York Co.**, Charters Settlement, 45.8395°N, 66.7391°W, 26.IX.2008, R.P. Webster // Mixed forest, in decaying (moldy) corncobs & cornhusks (1 ♂, RWC); same data as before but 2.V.2010 // Mixed forest opening, collected with net during evening flight between 16:30 & 20:00 h (1 ♂, HNHM).

####### Distribution in Canada and Alaska.

**NB (New Canadian record).** This Palaearctic species was not recognized by Casey (1889) in his monograph, unless as a synonym, but it was reported from North America the same year by Fauvel (1889) from MI and SC; the last record was by Ulke (1902) as the synonym *tenellus* (Erichson), from the District of Columbia (DC).

####### Natural history.

*Carpelimus gracilis* was collected by treading emergent *Carex* and grasses on a lake margin, sifted from decaying (moldy) corncobs and cornhusks and collected with an aerial net during evening flight between 16:30 & 20:00 h ADT in a mixed forest opening near a residential area. Adults were collected during May, June, and September.

###### Carpelimus lacustris

Taxon classificationAnimaliaColeopteraStaphylinidae

(Notman, 1924)

Figs 22–23

####### Material examined.

**Canada, New Brunswick, Albert Co.**, Caledonia Gorge P.N.A., 45.8380°N, 64.8484°W, 3.VII.2011, R.P. Webster // near Turtle Creek, old-growth hardwood forest, mossy seepage with some Carex, sifting saturated moss (1 ♂, RWC). **Charlotte Co.**, 3.0 km NW of Pomeroy Ridge, 45.3059°N, 67.4343°W, 5.VI.2008, R.P. Webster // Alder swamp, in moss hummocks with grasses (2 ♂, RWC). **Gloucester Co.**, near Acadian Historical Village, 47.7849°N, 65.0855°W, 23.V.2010, R.P. Webster // Salt marsh, treading *Spartina patens* and other grasses near tidal pool (1 ♂, HNHM). **Queens Co.**, ca. 3.5 km W of Lower Gagetown, 45.7497°N, 66.1846°W, 13.V.2008, R.P. Webster // Old red oak/red maple forest, in moist leaves on margin of vernal pond (1 ♂, RWC). **York Co.**, Kingsclear, Kelleys Creek at Sears Rd., 45.8723°N, 66.8414°W, 7.VI.2008, R.P. Webster // Alder swamp with red maple, in moist leaf & grass litter near pools (1 ♂, RWC); 8.5 km W of Tracy, off Rt. 645, 45.6821°N, 66.7894°W, 6.V.2008, R.P. Webster // Wet alder swamp, in moist litter & grass on hummocks near water [vernal pools] (2 ♂, RWC); 9.2 km W of Tracy, off Rt. 645, 45.6837°N, 66.8809°W, 22.V.2008, R.P. Webster // *Carex*, marsh adjacent to slow [flowing] stream, sifting grass litter near stream (1 ♂, HNHM; 1 ♂, RWC); same data but 2.V.2008 (1 ♀, HNHM).

####### Distribution in Canada and Alaska.

**NB (New Canadian record).** This species was described from Cranberry Lake, NY (Notman, 1924: 270). No other localities or data were included at the time and the species has not been reported again until now.

####### Natural history.

In NB, this species was associated with various wetland habitats. Specimens were sifted from saturated moss in a mossy seepage near a creek in a hardwood forest, found in moss hummocks with grasses in an alder swamp, sifted from moist leaves on a vernal pond margin in an old red oak and red maple forest, sifted from grass litter on muddy soil along a stream, in moist leaf and grass litter near vernal pools in a wet alder swamp, and sifted from grass litter near a slow-flowing stream. One specimen was collected in a salt marsh by treading *Spartina patens* and other grasses near tidal pool. Adults were collected from April to July.

**Figures 22–25.**
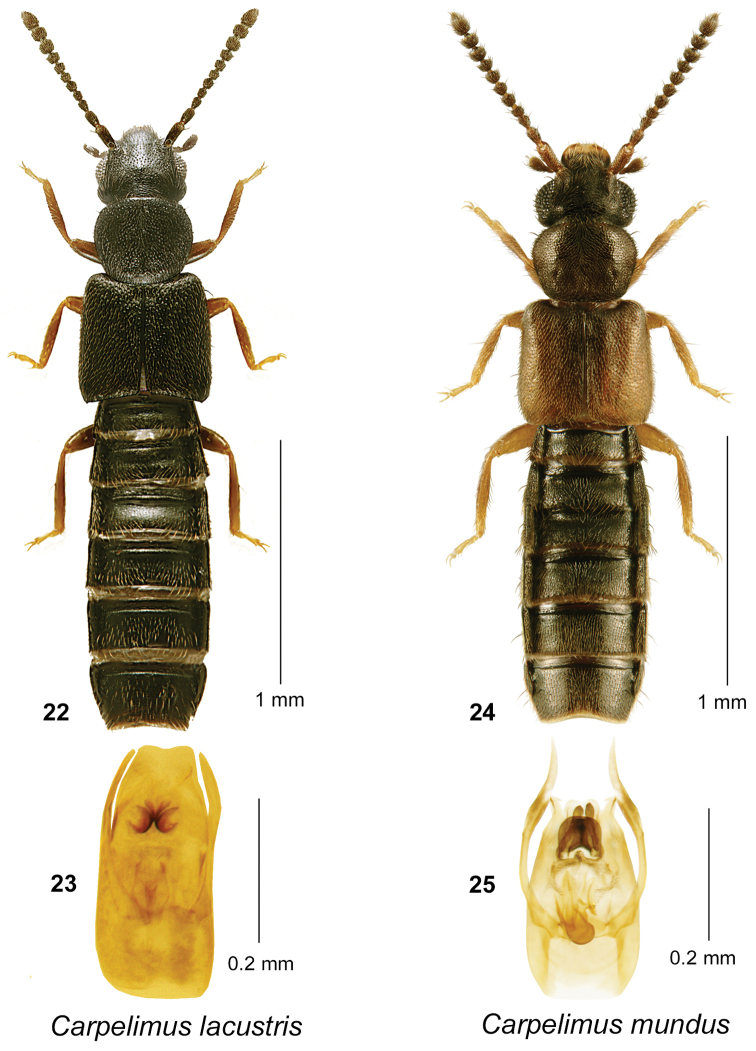
*Carpelimus lacustris* (Notman): **22** habitus in dorsal view **23** aedeagus in ventral view. *Carpelimus mundus* (Sharp): **24** habitus in dorsal view **25** aedeagus in ventral view.

###### Carpelimus mundus

Taxon classificationAnimaliaColeopteraStaphylinidae

(Sharp, 1876)†

Figs 24–25

####### Material examined.

**Canada, New Brunswick York Co.**, Charters Settlement, 45.8395°N, 66.7391°W, 12.VII.2006, 26.VIII.2007, 7.IX.2007, R.P. Webster, coll. // Mixed forest, u.v. light (1 ♂, HNHM; 2 ♂, 1 ♀, RWC); same locality data but 29.VIII.2007, R.P. Webster // Mixed forest, in decaying (moldy) corncobs & cornhusks (1 ♀, HNHM).

####### Distribution in Canada and Alaska.

**NB (New North American record).** The type locality is Ega (now Tefé, upper Amazon), Brazil (Sharp 1876: 397). Scheerpeltz (1933: 1086; catalog) added Argentina without details. Frank (1982) cited Ecuador. György Makranczy determined specimens of this species and thought that this was a highly unusual record for an essentially tropical species (all other members of this species group are also tropical).

####### Natural history.

Specimens from NB were collected at u.v. light in a residential yard near a mixed forest. One individual was collected from decaying (moldy) corncobs and cornhusks near a plastic composter.

###### Carpelimus obesus

Taxon classificationAnimaliaColeopteraStaphylinidae

(Kiesenwtter, 1844)† Removed from faunal list of NB

####### Note.

*Carpelimus obesus* was newly reported from NB by Klimaszewski et al. (2005) from the Acadia Research Forest in Sunbury Co. These specimens, which are in the AFC and LFC, were re-examined and are not *Carpelimus obesus*. *Carpelimus obesus* is larger and has different genitalia from any of the *Carpelimus* specimens captured during the above study. However, none of these specimens can be positively determined to species at this time.

###### Carpelimus probus

Taxon classificationAnimaliaColeopteraStaphylinidae

(Casey, 1889)

Figs 26–27

####### Material examined.

**Canada, New Brunswick, Carleton Co.**, Richmond, near Hovey Hill Protected [Natural] Area, 46.1152°N, 67.7632°W, 10.V.2005, R.P. Webster // Mixed forest with cedar, vernal pond in moist leaf litter on muddy soil (1 ♂, 1 ♀, HNHM; 1 ♂, RWC). **Queens Co.**, ca. 3.5 km W of Lower Gagetown, 45.7497°N, 66.1846°W, 13.V.2008, R.P. Webster // Old red oak/red maple forest, in moist leaves on margin of vernal pond (1 ♀, RWC). **Sunbury Co.**, Burton, W of Sunpoke Lake, 45.7590°N, 66.5778°W, 22.IV.2006, R.P. Webster // Red maple swamp, margin vernal pool in leaf litter (1 ♂, RWC). **York Co.**, Canterbury, “trail to Browns Mountain Fen”, 45.9033°N, 67.6260°W, 29.IV.2006, M. Giguère & R. Webster // Red maple swamp, in moist leaf litter near margin of vernal pond (1 ♂, HNHM; 1 ♂, RWC); 8.5 km W of Tracy, off Rt. 645, 45.6821°N, 66.7894°W, 6.V.2008, R.P. Webster // Wet alder swamp, in moist litter & grass on hummocks near water [vernal pools] (2 ♂, 2 ♀, RWC); 9.2 km W of Tracy, off Rt. 645, 45.6837°N, 66.8809°W, 22.V.2008, R.P. Webster // *Carex*, marsh adjacent to slow [flowing] stream, sifting grass litter near stream (1 ♂, RWC); 9.0 km W of Tracy, off Rt. 645, 45.6889°N, 66.8002°W, 5.IV.2010, R.P. Webster // Old beaver flowage, in grass litter on clay soil near small stream (1 ♂, RWC); Fredericton, Odell Park, 45.9539°N, 66.6666°W, 2-15.V.2013, C. Alderson & V. Webster // Hardwood stand, Lindgren funnel trap 1 m high under trees (1 ♂, RWC).

####### Distribution in Canada and Alaska.

**NB (New Canadian record).** The type locality was NC; the species has not been reported since.

####### Natural history.

This species was most commonly found among moist leaf litter on vernal pond margins in various forest types, including a mixed forest, red oak and red maple forest, red maple swamps, wet alder swamp, and a hardwood stand. A few were found among grass litter near slow flowing streams. Adults were collected during April and May.

**Figures 26–29.**
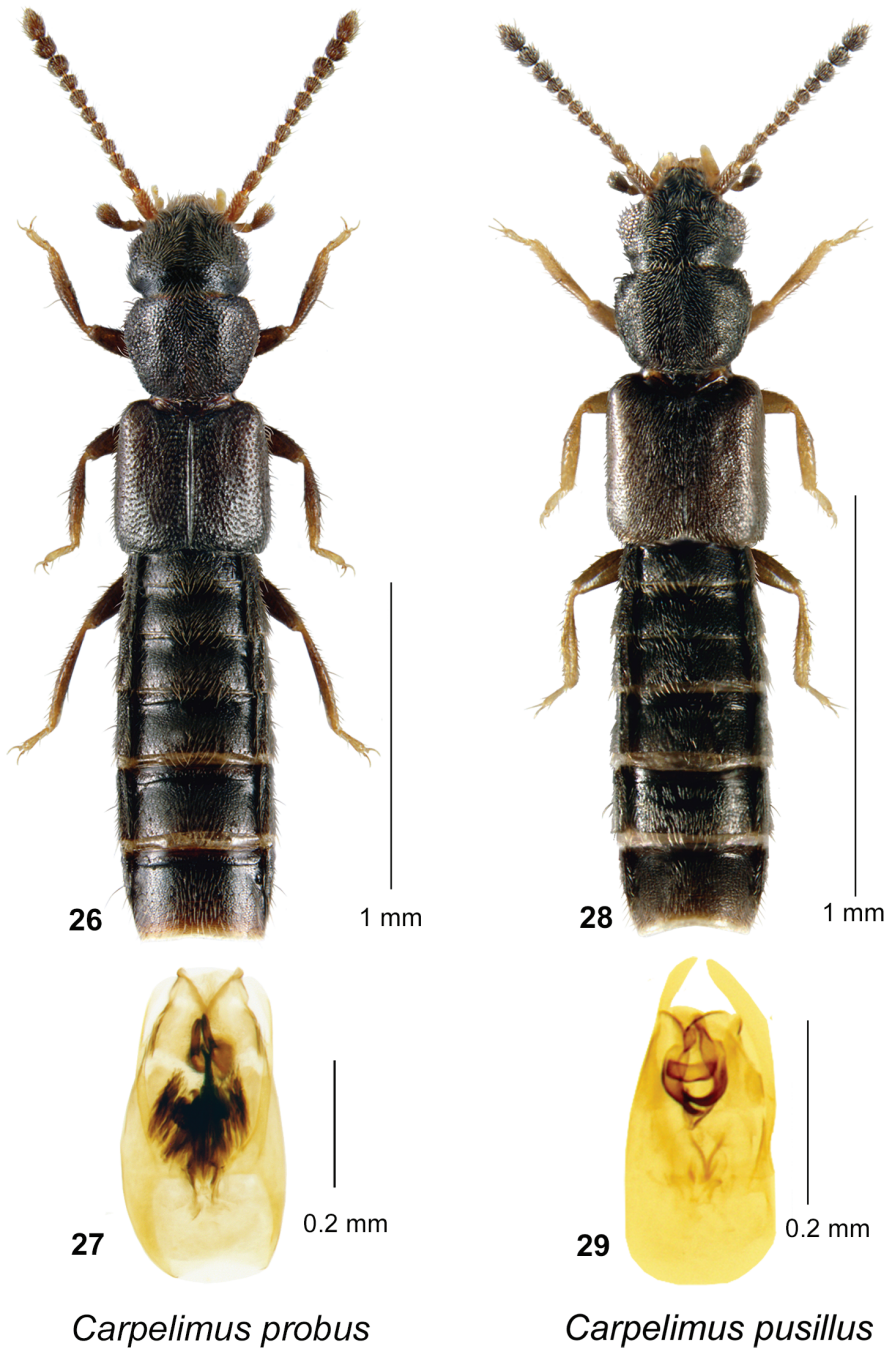
*Carpelimus probus* (Casey): **26** habitus in dorsal view **27** aedeagus in ventral view. *Carpelimus pusillus* (Gravenhorst): **28** habitus in dorsal view **29** aedeagus in ventral view.

###### Carpelimus pusillus

Taxon classificationAnimaliaColeopteraStaphylinidae

(Gravenhorst, 1802)†

Figs 28–29

####### Material examined.

**Canada, New Brunswick, Restigouche Co.**, Jacquet River Gorge P.N.A., 47.8207°N, 65.9961°W, 25.VI.2008, R.P. Webster, coll. // Black spruce bog, treading vegetation (1 ♂, HNHM). **York Co.**, Charters Settlement, 45.8395°N, 66.7391°W, 26.VII.2005, 9.VII.2006, 26.VII.2007, 26.VIII.2007, 7.IX.2007, 25.IX.2007, 3.IX.2010, R.P. Webster // Mixed forest, u.v. light (1 ♂, HNHM; 4 ♂, 4 ♀, 2 sex undetermined, RWC).

####### Distribution in Canada and Alaska.

**NB (New Canadian record).** This Palaearctic species was first reported in North America by Fauvel (1871) and Fauvel (1889) from MA, MI, and TX in the USA, distribution typical for an adventive species; he also included LeConte’s record of *subtilis* (Erichson) from the “southern and western states”, which LeConte confirmed; the last record of *pusillus* in North America was by Ulke (1902) from DC.

####### Natural history.

All but one of the specimens known from NB were collected at a u.v. light in a mixed forest in July, August, and September. One specimen was collected by treading vegetation in a black spruce bog in June.

###### Carpelimus quadripunctatus

Taxon classificationAnimaliaColeopteraStaphylinidae

(Say, 1831)

Figs 30–31

####### Material examined.

**New Brunswick, Queens Co.**, Bayard near Nerepis River, 45.4442°N, 66.3292°W, 25.V.2008, R.P. Webster // Pond margin, in moist grass litter on mud (1 ♂, 1 ♀, 1 sex undetermined, RWC); Welsford near Nerepis River, 45.4441°N, 66.3300°W, 27.VI.2006, R.P. Webster, coll. // Margin of oxbow, treading emergent grass into water (1 ♂, RWC). **Sunbury Co.**, Maugerville, Portobello Creek N.W.A., 45.8882°N, 66.4248°W, 16.VII.2004, R.P. Webster // Silver maple swamp, margin of river under litter on muddy soil (1 ♂, RWC). **York Co.** Fredericton at Saint John River, 45.9598°N, 66.6258°W, 4.VII.2004, 19.VII.2005, R.P. Webster // River margin under drift material (1 ♀, HNHM; 1 sex undetermined, RWC); Kingsclear, Mazerolle Settlement, 45.8729°N, 66.8311°W, 28.IV.2006, R.P. Webster, coll. // Margin of stream (sun-exposed), in fine gravel/sand near water (2 ♂, RWC); Keswick River at Rt. 105, 45.9938°N, 66.8344°W, 3.VI.2008, R.P. Webster // Silver maple swamp near river margin, in leaf and grass litter on mud/clay soil (1 ♂, 1 ♀, RWC).

####### Distribution in Canada and Alaska.

ON, QC, **NB** (Bousquet et al. 2013).

####### Natural history.

This species was found on pond, stream, and river margins in moist grass litter on mud, in leaf and grass litter on mud and clay soil, under litter on muddy soil, under drift material, and in fine gravel/sand on a stream margin close to water. One individual was collected by treading emergent grass into water along the margin of an oxbow. Adults were collected during May, June, and July.

**Figures 30–33.**
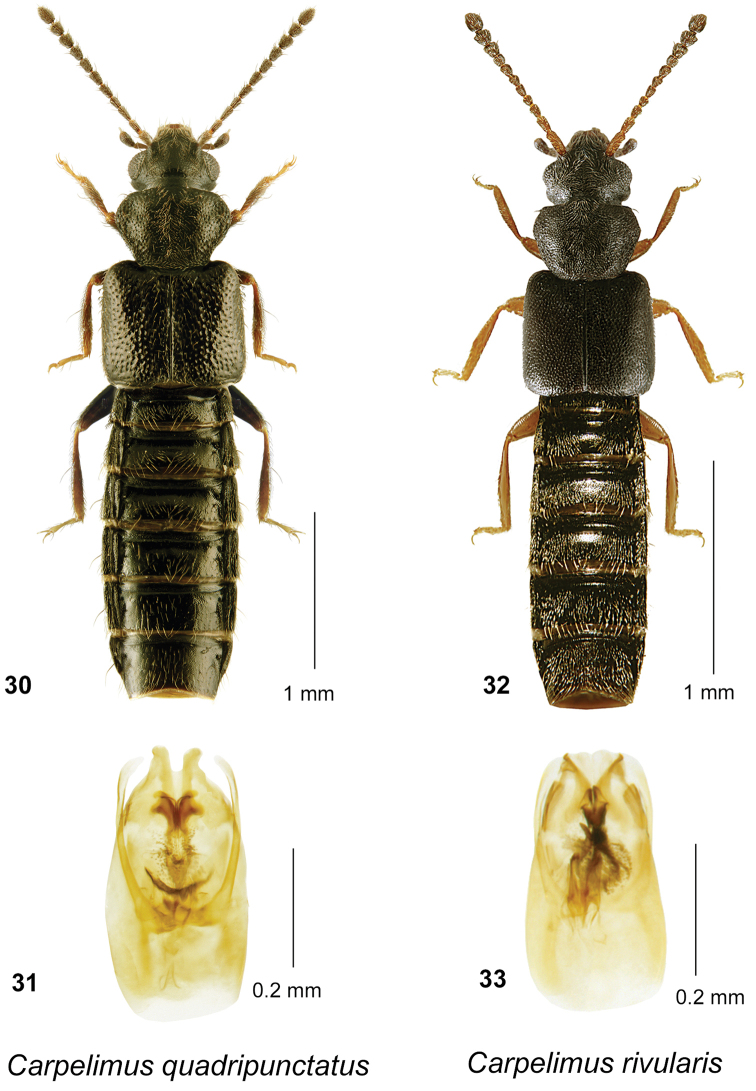
*Carpelimus quadripunctatus* (Say): **30** habitus in dorsal view **31** aedeagus in ventral view. *Carpelimus rivularis* (Motschulsky) **32** habitus in dorsal view **33** aedeagus in ventral view.

###### Carpelimus rivularis

Taxon classificationAnimaliaColeopteraStaphylinidae

(Motschulsky, 1860)†

Figs 32–33

####### Material examined.

**New Brunswick, Queens Co.**, Bayard near Nerepis River, 45.4442°N, 66.3292°W, 25.V.2008, R.P. Webster // Pond margin, in moist grass litter on mud (1 ♂, HNHM; 2 ♂, RWC). **Saint John Co.**, Musquash, 45.1837°N, 66.3376°W, 7.V.2006, R.P. Webster, coll. // Inland margin of salt marsh, in litter on muddy soil (1 ♂, HNHM; 4 ♂, RWC). **York Co.**, Keswick River at Rt. 105, 45.9938°N, 66.8344°W, 3.VI.2008, R.P. Webster // Silver maple swamp near river margin, in leaf and grass litter on mud/clay soil (1 ♂, RWC).

####### Distribution in Canada and Alaska.

**NB (New Canadian record).** *Carpelimus spretus* was synonymized with the Palaearctic *Carpelimus rivularis* (Motschulsky) by Bernhauer and Schubert (1911) and it was cited as such by Schülke and Smetana (2015: 784), although Downie and Arnett (1996) cited *Carpelimus spretus* as a valid species with the type localities (MD, NC, and PA). They also cited *Carpelimus rivularis* as “poorly known”, but with two of the same localities, PA, MD (p. 443). The two species are clearly different and are treated as such here. Notman (1920) recorded *rivularis* from NY. Hatch (1957) further complicated the matter by including *Carpelimus rivularis* as a synonym of *Carpelimus bilineatus* Stephens from the Pacific Northwest.

####### Natural history.

Specimens were found on the inland margin of a salt marsh in litter on muddy soil, in moist grass litter on mud on a pond margin near a river, and in leaf and grass litter on mud/clay soil near a river margin in a silver maple swamp. Adults were collected during May and June.

###### Carpelimus spretus

Taxon classificationAnimaliaColeopteraStaphylinidae

(Casey, 1889)

Figs 34–35

####### Material examined.

**Canada, New Brunswick, Carleton Co.**, Hartland, Hwy 2 at St. John River, 46.3136°N, 67.5376°W, 2.VIII.2004, R.P. Webster // River margin, on moist clay soil among tall grass (1 sex undetermined, RWC). Hartland, Middle Becaguimec Island, 46.3038°N, 67.5333°W, 23.VI.2006, R. Capozi & R. Webster // Margin of Saint John River, among cobblestones near water (1 ♀, RWC); Belleville, Meduxnekeag Valley Nature Preserve, 46.1942°N, 67.6832°W, 2.VI.2008, 9.VI.2008, R.P. Webster // River margin, among small cobblestones set in sand and fine gravel near water’s edge (1 ♂, 1 ♀, HNHM; 1 ♂, 1 ♀, RWC); same locality but 46.1931°N, 67.6825°W, 31.V.2005, R.P. Webster, coll. // river margin under drift material (1 ♂, HNHM). **Queens Co.**, Grand Lake at Whites Cove, 45.86795°N, 66.06415°W, 4.VIII.2005, R.P. Webster // Lake margin, cobblestone beach, under cobblestones (1 ♂, RWC). **York Co.**, 1.5 km N of Durham Bridge, 46.1408°N, 66.6179°W, 15.VI.2008, R.P. Webster // Nashwaak River, river margin, among cobblestones near outflow of brook 1 ♂, 2 ♀, RWC).

####### Distribution in Canada and Alaska.

**NB (New Canadian record).** See comments above regarding *Carpelimus spretus* and the Palaearctic *Carpelimus rivularis*. Downie and Arnett (1996) cited *Carpelimus spretus* as a valid species with the type localities (MD, NC, and PA). Ulke (1902) recorded *Carpelimus spretus* from DC.

####### Natural history.

*Carpelimus spretus* were collected along river margins in NB. Adults were typically found among cobblestones near water’s edge. Adults were collected in June and August.

**Figures 34–37.**
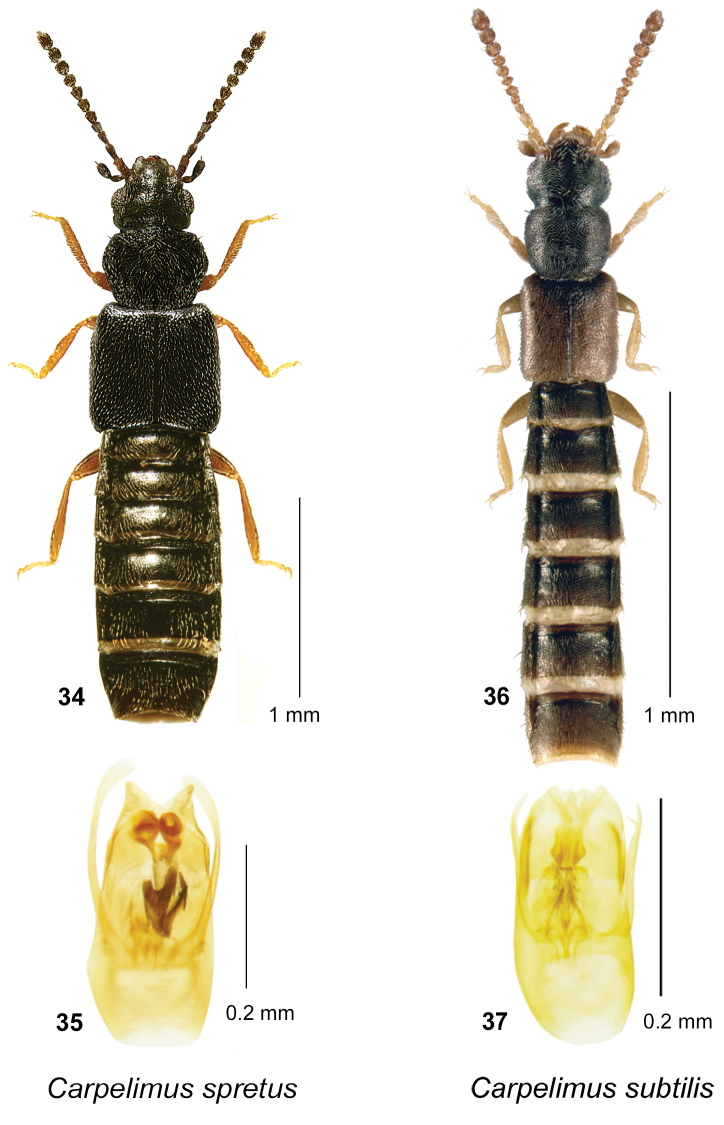
*Carpelimus spretus* (Casey): **34** habitus in dorsal view **35** aedeagus in ventral view. *Carpelimus subtilis* (Erichson): **36** habitus in dorsal view **37** aedeagus in ventral view.

###### Carpelimus subtilis

Taxon classificationAnimaliaColeopteraStaphylinidae

(Erichson, 1839)†

Figs 36–37

####### Material examined.

**New Brunswick, Restigouche Co.**, Dionne Brook P.N.A., 47.9030°N, 68.3503°W, 15-27.VI.2011, M. Roy & V. Webster // Old-growth northern hardwood forest, Lindgren funnel trap (1 ♂, RWC). **York Co.**, Keswick River at Rt. 105, 45.9938°N, 66.8344°W, 3.VI.2008, R.P. Webster // Silver maple swamp near river margin, in leaf and grass litter on mud/clay soil (2 ♂, 1 ♀ HNHM; 5 ♂, 3 ♀, 2 sex undermined, RWC); Douglas, Currie Mountain, 45.9844°N, 66.7592°W, 3-15.V.2013, C. Alderson & V. Webster // Mixed forest with *Quercus rubra*, Lindgren funnel trap 1 m high under *Quercus rubra* (1 ♂, AFC).

####### Distribution in Canada and Alaska.

**NB**, NS (Bousquet et al. 2013). This Palaearctic species was first reported in North America by LeConte (1877), but this was a misidentification of *Carpelimus pusillus* (q. v.); Ulke (1902) recorded *Carpelimus subtilis* from DC, and Bernhauer and Schubert (1911) listed it from PA and RI, the type locality of Casey’s synonym, *Carpelimus indigens*.

####### Natural history.

Casey (1889) reported *Carpelimus indigens* as gregarious, on the underside of a stone in the damp bottom of a partially dry ditch. Most NB specimens were sifted from leaf and grass litter on mud/clay soil near a river margin in a silver maple swamp. Two specimens were captured in Lindgren funnel traps in an old-growth northern hardwood forest and a mixed forest. Adults were collected during April, May, and June.

###### Carpelimus weissi

Taxon classificationAnimaliaColeopteraStaphylinidae

(Notman, 1924)

Figs 38–39

####### Material examined.

**Canada, New Brunswick, Charlotte Co.**, 5.0 km NW of Pomeroy Ridge, 45.3059°N, 67.4343°W, 5.VI.2008, R.P. Webster // Alder swamp, in moss hummocks with grasses (1 ♂, HNHM; 1 ♂, 1 ♀, RWC); 5.2 km NW of Pomeroy Ridge, 45.3087°N, 67.4362°W, 5.VI.2008, 16.VI.2008, R.P. Webster // Red maple swamp, in sphagnum with grasses near vernal pools (1 ♀ HNHM; 1 ♂, 5 ♀, 1 sex undetermined, RWC); 3 km SW of King Brook Lake, 45.3194°N, 67.4414°W, 27.V.2007, R.P. Webster // Wet eastern white cedar, red maple & black ash swamp, in moist litter & moss near small pool (1 ♂, RWC).

####### Distribution in Canada and Alaska.

**NB (New Canadian record).** There are no other records of this species aside from the unique type from NJ.

####### Natural history.

*Carpelimus weissi* specimens were sifted from moss in moss hummocks, sphagnum near vernal pools, and from moist litter and moss near vernal pools in alder swamps, red maple swamps, and an eastern white cedar swamp with red maple and black ash (*Fraxinus nigra* Marsh), respectively. Adults were collected during May and June (NB) and August (type).

**Figures 38–41.**
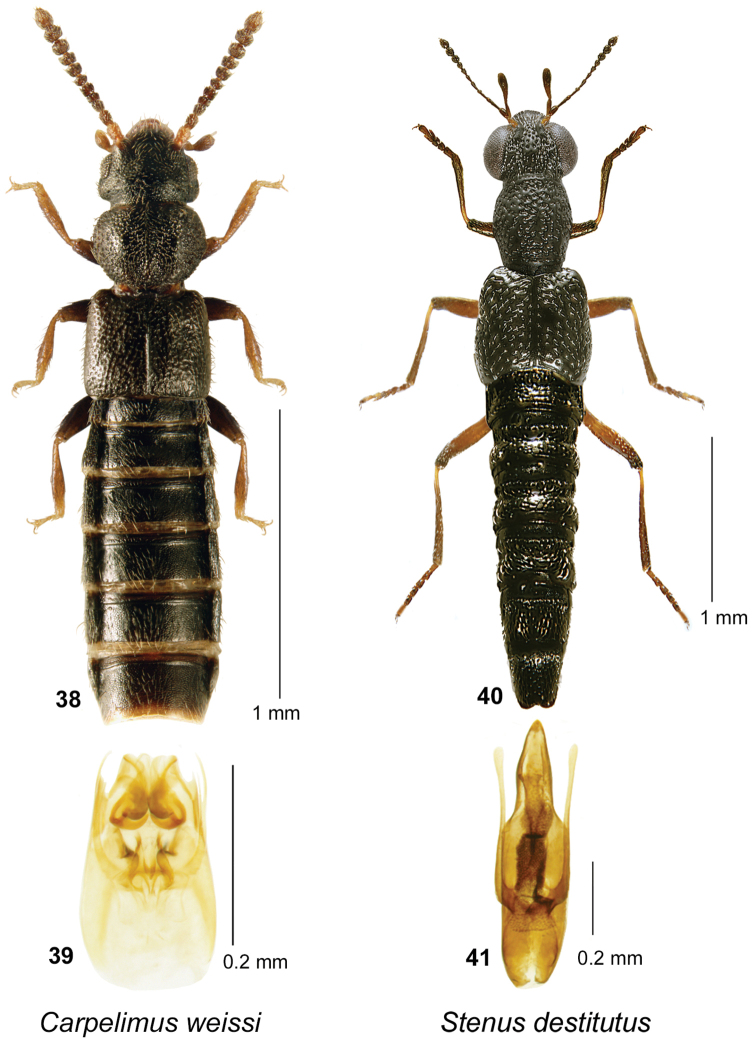
*Carpelimus weissi* (Notman): **38** habitus in dorsal view **39** aedeagus in ventral view. Stenus (Hypostenus) destitutus Puthz: **32** habitus in dorsal view **33** aedeagus in ventral view.

###### Ochthephilus planus

Taxon classificationAnimaliaColeopteraStaphylinidae

(LeConte, 1861)

####### Additional records.

**New Brunswick, Madawaska Co.**, Gagné Brook at First Lake, 47.6077°N, 68.2534°W, 23.VI.2010, M. Turgeon & R. Webster // northern hardwood forest, shaded brook, among gravel on gravel bar, splashing and turning gravel (1 ♂, HNHM; 1 ♂, RWC). **Restigouche Co.**, Jacquet River Gorge P.N.A. 47.8010°N, 66.0963°W, 15.VI.2009, R. P. Webster // Cold shaded brook, under cobblestone near brook margin (1 sex undetermined, RWC); Mount Atkinson, 447 m elev., 47.8192°N, 68.2618°W, 21.VII.2010, R.P. Webster // Boreal forest, small shaded spring-fed brook with mossy margin, sifting saturated moss (3 ♂, 1 ♀, RWC).

####### Distribution in Canada and Alaska.

AK, YT, BC, AB, ON, QC, NB, NF (Bousquet et al. 2013, Makranczy 2014). Makranczy (2014) first recorded this species from NB on the basis of the Gagné Brook record.

####### Natural history.

Adults of *Ochthephilus planus* were found along cold-shaded brooks in northern hardwood, mixed, and boreal forests in NB. Specimens were found in gravel, under cobblestones, and in saturated moss. Adults were collected in June and July.

###### Platystethus (Craetopycrus) degener

Taxon classificationAnimaliaColeopteraStaphylinidae

Mulsant & Rey, 1878†

####### Material examined.

**New Brunswick, York Co.** Keswick Ridge, 45.9962°N, 66.8781°W, 13-28.VIII.2014, C. Alderson & V. Webster // Field/meadow, Lindgren funnel trap (1, RWC).

####### Distribution in Canada and Alaska.

ON, QC, **NB** (Bousquet et al. 2013).

####### Natural history.

One individual of this adventive species was captured in a Lindgren funnel trap.

###### Thinodromus corvinus

Taxon classificationAnimaliaColeopteraStaphylinidae

(Casey, 1889)

####### Material examined.

**New Brunswick, Carleton Co.**, Jackson Falls, Bell Forest, 46.2200°N, 67.7231°W, 1-8.VI.2009, R.P. Webster & M.-A. Giguère // Rich Appalachian hardwood forest with some conifers, Lindgren funnel trap (1 sex undetermined, RWC); same locality data, forest type, and trapping method but 7-21.VI.2012, C. Alderson & V. Webster (1 sex undetermined, RWC).

####### Distribution in Canada and Alaska.

ON, QC, **NB** (Bousquet et al. 2013).

####### Natural history.

Both specimens from NB were captured in Lindgren funnel traps in a hardwood forest.

#### Subfamily Scydmaeninae, Leach 1815

Members of this subfamily occur in forest litter, moss, rotting logs, tree holes, and other moist habitats such as marshes and bogs (O’Keefe 2000). O’Keefe (2000) should be consulted for details on the adult and larval morphology, biology, and classification of this subfamily. Adults are predators of oribatid mites (Schuster 1966, Schmid 1988). Bouchard et al. (2013) listed 49 species of Scydmaeninae from Canada and eight species for NB. However, many genera of this subfamily in North America need to be revised, and a number of undescribed species are known from NB and Canada. Here, we add another species to the faunal list of the province.

##### Supertribe Scydmaenitae Leach, 1815

###### Tribe Glandulariini Schaufuss, 1889

####### Brachycepsis subpunctata

Taxon classificationAnimaliaColeopteraStaphylinidae

(LeConte, 1852)

######## Material examined.

**New Brunswick, Carleton Co.**, Jackson Falls, Bell Forest, 46.2200°N, 67.7231°W, 4-12.VI.2008, 12-19.VI.2008, 19-27.VI.2008, 28.VII-6.VIII.2008, R.P. Webster // Rich Appalachian hardwood forest with some conifers, Lindgren funnel trap (9 sex undetermined, RWC); 15 km W of Tracy off Rt. 645, 45.6848°N, 66.8821°W, 25.IV-4.V.2009, R.P. Webster & M.-A. Giguère // Old red pine forest, Lindgren funnel trap (1 sex undetermined, RWC); Canterbury, Eel River P.N.A., 45.8967°N, 67.6343°W, 12-25.VIII.2014, C. Alderson & V. Webster // Old-growth eastern white cedar swamp & fen, Lindgren funnel traps (1 sex undetermined, RWC).

######## Distribution in Canada and Alaska.

ON, QC, **NB**, NS, PE, LB, NF (Bousquet et al. 2013).

######## Natural history.

*Brachycepsis subpunctata* adults were captured in Lindgren funnel traps in a rich Appalachian hardwood forest, an old red pine forest, and an old-growth eastern white cedar swamp and fen. Specimens were collected in April, May, June, and August in NB.

#### Subfamily Steninae MacLeay, 1825

Members of this subfamily occur in various habitats, especially wetland habitats where they occur on rocks and plants near streams and rivers, ponds, and marshes (Newton et al. (2000). They also can be found on vegetation away from water and in forest leaf litter and debris. Adults are specialized predators of Collembola and other small arthropods (Newton et al. 2000). Adults use a specialized protrusible labium for prey capture and possess special pygidial glands that allow them to skim across water surfaces (Jenkins 1960, Betz 1996, 1998, 1999). The subfamily includes two genera, *Dianous* and *Stenus*, in North America, with two species of *Dianous* and 112 *Stenus* species reported from Canada (Bousquet et al. 2013), including one species of *Dianous* and 45 species of *Stenus* from NB. In this account, we record one additional species of *Dianous* and 13 additional *Stenus* species from the province. Stenus (Hypostenus) destitutus Puthz is newly recorded for Canada.

##### Dianous nitidulus

Taxon classificationAnimaliaColeopteraStaphylinidae

LeConte, 1874

###### Material examined.

**New Brunswick, Albert Co.**, Caledonia Gorge P.N.A., 45.7930°N, 64.7764°W, 1.VII.2011, R. P. Webster //Small rocky clear-cold river [Caledonia Brook], splashing exposed rocks covered with moss in middle of river (1 ♀, NBM); Caledonia Gorge P.N.A., 45.7808°N, 64.7775°W, 4.VII.2011, R.P. Webster // Canada Creek, cold-clear, shaded rocky brook with small waterfalls, sifting saturated moss on rocks near flowing water (1 ♀, NBM); Caledonia Gorge P.N.A., 45.8432°N, 64.8411°W, 5.VII.2011, R.P. Webster // Turtle Creek, rocky, cool-water, shaded creek, in saturated moss on rocks 1 ♂, NBM); Caledonia Gorge P.N.A., 45.7985°N, 64.7755°W, 18.VIII.2012, R.P. Webster // Crooked Creek near Caledonia Brook, splashing sun-exposed moss covered rocks (1 ♂, NBM); Caledonia Gorge P.N.A., 45.7706°N, 64.8063°W, 2.VII.2011, 12.IX.2012, R.P. Webster // McKinley Brook, in moss on rocks in shaded brook (2 ♀, NBM). **Carleton Co.**, Belleville, Meduxnekeag Valley Nature Preserve, 46.1895°N, 67.6704°W, 11.VI.2010, R.P. Webster // Rich Appalachian hardwood forest, margin of shaded spring-fed brook near small waterfall (2 sex undetermined, RWC). **Madawaska Co.**, Gagné Brook at First Lake, 47.6077°N, 68.2534°W, 23.VI.2010, M. Turgeon & R. Webster // Northern hardwood forest, shaded brook, among gravel on gravel bar, splashing and turning gravel (1 ♂, NBM); at Green River, 47.6918°N, 68.3202°W, 21.VI.2010, M. Turgeon & R. Webster // River margin, among gravel on gravel bar (1 ♂, NBM); Jalbert Brook, 262 m elev., 47.6470°N, 68.3026°W, 23.VI.2010, R.P. Webster // Old-growth mixed forest, shaded brook, on gravel on gravel bar (1 sex undetermined, RWC). **Restigouche Co.**, Jacquet River Gorge P.N.A. 47.8010°N, 66.0963°W, 15.VI.2009, 24.V.2010, R. P. Webster // Cold shaded brook, on rocks or in moss on rocks on brook margin or within brook (5 sex undetermined, NBM; 1, ♂, 1 ♀, 5 sex undetermined, RWC); same locality and collector, 47.8257°N, 66.0779°W, 24.V.2010 // Partially shaded cobblestone bar near outflow of brook at Jacquet River, under cobblestones & gravel on sand (1, NBM); Kedgwick Forks, 47.9085°N, 67.9057°W, 23.VI.2010, R. P. Webster // River margin, on clay/sand under alders (1 ♀, NBM).

###### Distribution in Canada and Alaska.

AK, YT, BC, AB, SK, QC, **NB**, NS, NF (Bousquet et al. 2013).

###### Natural history.

In NB, most specimens of *Dianous nitidulus* were found along fast-flowing, cold, shaded brooks, shaded streams, and shaded river margins. Adults occurred on rocks or in moss (often saturated with water) on rocks on the stream margin and within the streams themselves. Some individuals were found on gravel bars or on clay/sand along shaded brooks and river margins. Adults were collected by splashing moss, rocks, and gravel in the above habitats, in May, June, July, and September.

##### Stenus (Hemistenus) sibiricus

Taxon classificationAnimaliaColeopteraStaphylinidae

J.R. Sahlberg, 1880

###### Material examined.

**New Brunswick, Restigouche Co.**, Jacquet River Gorge P.N.A. 47.8200°N, 66.0015°W, 13.V.2010, R. P. Webster // Under alders in leaf litter & moss near small brook in *Carex* marsh (1 ♀, RWC).

###### Distribution in Canada and Alaska.

AK, YT, NT, BC, AB, SK, MB, ON, QC, **NB**, NF (Bousquet et al. 2013).

###### Natural history.

The single NB specimen of this species was sifted from leaf litter and moss under alders near a small brook.

##### Stenus (Hypostenus) alexanderi

Taxon classificationAnimaliaColeopteraStaphylinidae

Puthz, 1971

###### Material examined.

**New Brunswick, Charlotte Co.**, near New River, 45.21176°N, 66.61790°W, 7.V.2007, R.P. Webster // Small pond & marsh, treading litter & moss into water (1 ♂, RWC).

###### Distribution in Canada and Alaska.

BC, AB, MB, ON, QC, **NB**, NF (Bousquet et al. 2013).

###### Natural history.

The single NB specimen of *Stenus alexanderi* was collected by treading litter and moss into water on the margin of a small pond/marsh.

##### Stenus (Hypostenus) destitutus

Taxon classificationAnimaliaColeopteraStaphylinidae

Puthz, 2001

Figs 40–41

###### Material examined.

**Canada, New Brunswick, Albert Co.**, Caledonia Gorge P.N.A., 45.7930°N, 64.7764°W, 1.VII.2011, R.P. Webster // Small rocky clear cold river (Caledonia Creek), splashing exposed rocks with moss in middle of river (2 ♂, 2 ♀, RWC); same locality and collector but 45.7935°N, 64.7744°W, 22.V.2012 // Crooked Creek, cold clear rocky stream in *Carex* hummock in stream (1 ♂, RWC). **Saint John Co.**, Fundy Trail Parkway, 45.4227°N, 65.4110°W, 23.VIII.2006, R.P. Webster // Margin of Big Salmon River, among gravel & cobblestones near water (1 ♀, RWC); same locality and collector but 45.4222°N, 65.4052°W, 17.VII.2010 // River margin in emergent *Carex* hummocks in flowing water (2 ♂, 2 sex undetermined, RWC).

###### Distribution in Canada and Alaska.

**NB (New Canadian record).** This species was previously known from as far north as NY and NH.

###### Natural history.

In NB, adults of *Stenus destitutus* were found along clear, cold, fast-flowing river and stream margins. Most specimens were collected by splashing exposed rocks with moss in the middle of a river or splashing emergent *Carex* hummocks within streams. One specimen was found among gravel and cobblestones near water. Adults were collected in May, July, and August.

##### Stenus (Hypostenus) punctatus

Taxon classificationAnimaliaColeopteraStaphylinidae

Erichson, 1840

###### Material examined.

**New Brunswick, Charlotte Co.**, near New River, 45.21176°N, 66.61790°W, 7.VII.2006, 22.IX.2006, R.P. Webster // Eastern white cedar swamp, small pond & marsh, treading *Carex* hummocks into water (2 ♂, RWC). **Queens Co.**, Grand Lake Meadows P.N.A., 45.8227°N, 66.1209°W, 5.VII.2010, R.P. Webster // Old silver maple forest & seasonally flooded marsh, treading (1 ♂, RWC); C.F.B. Gagetown, 45.7516°N, 66.1866°W, 20.V-4.VI.2015, C. Alderson & V. Webster // Old mixed forest with *Quercus rubra*, Lindgren funnel trap in canopy (1 ♂ , RWC).

###### Distribution in Canada and Alaska.

ON, QC, **NB** (Bousquet et al. 2013).

###### Natural history.

In NB, this species was found in an eastern white cedar swamp in a small pond and marsh, a seasonally flooded marsh, and an old mixed forest. Specimens were collected by treading *Carex* hummock into water, treading marsh vegetation, and one was captured in a Lindgren funnel trap. Adults were collected in May, June, July, and September.

##### Stenus (Stenus) carinicollis

Taxon classificationAnimaliaColeopteraStaphylinidae

Casey, 1884

###### Material examined.

**New Brunswick, Albert Co.**, Caledonia Gorge P.N.A., 45.7682°N, 64.8092°W, 30.VI.2011, R.P. Webster // Spruce & balsam fir forest near small brook, sifting litter (1 ♂, RWC); Caledonia Gorge P.N.A., 45.8432°N, 64.8411°W, 5.VII.2011, R.P. Webster // Turtle Creek, in rotten log (1 ♀, RWC).

###### Distribution in Canada and Alaska.

ON, QC, **NB**, NS, NF (Bousquet et al. 2013).

###### Natural history.

The NB specimens were sifted from litter near a small brook in a spruce and balsam fir forest and a rotten log near a creek in June and July.

##### Stenus (Stenus) comma comma

Taxon classificationAnimaliaColeopteraStaphylinidae

LeConte, 1863*

###### Material examined.

**New Brunswick, Restigouche Co.**, Little Tobique River near Red Brook, 47.4465°N, 67.0689°W, 13.VI.2006, R.P. Webster // River margin, under debris on clay sand/mix (3 sex undetermined, RWC); Jacquet River Gorge P.N.A., 47.8257°N, 66.0780°W, 24.V.2010 R. P. Webster // margin of Jacquet River, clay bank on bare clay (3 sex undetermined , NBM; 1 sex undetermined, RWC); Sport Camp Brook, 47.9582°N, 68.0183°W, 30.VII.2012, R.P. Webster & M. Turgeon // Logging road through spruce & cedar forest, on mud/clay of dried puddle on roadside (1 sex undetermined RWC). **York Co.**, Keswick River at Rt. 105, 45.9943°N, 66.8337°W, 18.VI.2004, R.P. Webster // River margin, splashing on clay/sand mix on steep bank (5 sex undetermined, RWC).

###### Distribution in Canada and Alaska.

AK, YK, NT, BC, AB, SK, MB, ON, QC, **NB** (Bousquet et al. 2013).

###### Natural history.

Most adults of *Stenus comma comma* were found along river margins on (often steep) clay banks, on bare clay, under debris on clay/sand mix, and by splashing clay/sand mix on a steep bank. One individual was found on mud/clay in a dried puddle on a logging road. Specimens were collected during May, June, and July.

##### Stenus (Stenus) difficilis

Taxon classificationAnimaliaColeopteraStaphylinidae

Casey, 1884

###### Material examined.

**New Brunswick, Saint John Co.**, Dipper Harbour, 45.1169°N, 66.3771°W, 7.VII.2006, R.P. Webster // Margin of salt marsh, in seepage area, treading (2 ♂, 1 ♀, RWC); same locality and collector but 45.1182°N, 66.3790°W, 28.V.2010 // Upper margin of salt marsh, in grass litter (sifted) in seepage area with *Carex* & *Spartina patens* (1 ♂, 2 ♀, RWC).

###### Distribution in Canada and Alaska.

AB, SK, ON, QC, **NB** (Bousquet et al. 2013).

###### Natural history.

In NB, *Stenus difficilis* was found along the margins of salt marshes. Specimens were collected in grass litter in seepage areas with *Carex* and *Spartina patens* (Ait.) Muhl. (salt-meadow grass) by treading or sifting vegetation. Adults were collected in May and July.

##### Stenus (Stenus) egenulus

Taxon classificationAnimaliaColeopteraStaphylinidae

Puthz, 1988

###### Material examined.

**New Brunswick, Carleton Co.**, Wakefield [Belleville], Meduxnekeag Valley Nature Preserve, 46.1931°N, 67.6825°W, 13.VII.2004, R.P. Webster // River margin, under drift material (1 ♂, RWC). **Restigouche Co.**, Jacquet River Gorge P.N.A. 47.8010°N, 66.0962°W, 15.VIII.2010, R. P. Webster // River margin, on mud (1 ♀, RWC); Wild Goose Lake, 420 m elev., 47.8540°N, 68.3219°W, 7.VI.2011, R.P. Webster // Lake margin with emergent *Carex* and grasses, treading *Carex* and grasses (1 ♂, RWC).

###### Distribution in Canada and Alaska.

AK, YK, NT, BC, AB, SK, MB, ON, QC, **NB**, LB, NF (Bousquet et al. 2013).

###### Natural history.

One individual of *Stenus egenulus* was found under drift material on a river margin, another on mud along a river margin, and one was collected by treading in an area with emergent *Carex* and grasses along a lake margin. Adults were collected in June, July, and August.

##### Stenus (Stenus) fulvoguttatus

Taxon classificationAnimaliaColeopteraStaphylinidae

Notman, 1920

###### Material examined.

**New Brunswick, York Co.**, Fredericton, 45.9361°N, 66.6747°W, 17.VIII.2009, R.P. Webster // Beaver dam, outer margin under overhanging sticks near water (1 ♂, RWC); Charters Settlement, 45.8456°N, 66.7267°W, 1.V.2010, 5.V.2010, 10.VI.2010, R.P. Webster // Beaver dam, among sticks and debris near an overflow area of dam (near flowing water) (2 ♂, 2 ♀, RWC).

###### Distribution in Canada and Alaska.

ON, QC, **NB** (Bousquet et al. 2013).

###### Natural history.

All specimens of *Stenus fulvoguttatus* from NB were found in beaver (*Castor canadensis* Kuhl) dams. Adults were found on the outer margin of the dams under overhanging sticks and among sticks and debris near overflow areas of the dam. Specimens were collected in June and August.

##### Stenus (Stenus) pluto

Taxon classificationAnimaliaColeopteraStaphylinidae

Casey, 1884

###### Material examined.

**New Brunswick, Albert Co.**, Caledonia Gorge P.N.A., 45.7930°N, 64.7764°W, 1.VII.2011, R.P. Webster // Small rocky clear cold river margin (Caledonia Creek), sifting drift material (tree bud material) near eddy area (1 ♂, RWC). **Restigouche Co.**, Jacquet River Gorge P.N.A. 47.8256°N, 66.0770°W, 13.VIII.2010, R. P. Webster // Large shaded brook among cobblestones (1 ♀, RWC); Wild Goose Lake, 420 m elev., 47.8540°N, 68.3219°W, 7.VI.2011, R.P. Webster // Lake margin with emergent *Carex* and grasses, treading *Carex* and grasses (5 ♂, 2 ♀, RWC); Summit Lake, 47.7825°N, 68.3199°W, 7.VI.2011, R.P. Webster // Lake margin, *Carex* marsh, treading *Carex* hummocks and emergent vegetation (1 ♂, RWC).

###### Distribution in Canada and Alaska.

BC, SK, MB, ON, QC, **NB** (Bousquet et al. 2013).

###### Natural history.

Most specimens of *Stenus pluto* were found along lake margins (two sites) with emergent vegetation (*Carex*, *Carex* hummocks, and grasses). Adults were collected by treading vegetation into water. One specimen was sifted from drift material (tree bud material) near an eddy area along a small rocky, clear, cold river margin and another was found among cobblestones along a large shaded brook. This species was collected in June, July, and August in NB.

##### Stenus (Stenus) pumilio

Taxon classificationAnimaliaColeopteraStaphylinidae

Erichson, 1839

###### Material examined.

**New Brunswick, Charlotte Co.**, near New River, 45.2118°N, 66.6179°W, 13.VI.2008, R.P. Webster // Sedge marsh, treading sphagnum and *Carex* hummock into water (1 ♀, RWC).

###### Distribution in Canada and Alaska.

AK, YK, NT, MB, ON, QC, **NB** (Bousquet et al. 2013).

###### Natural history.

The sole specimen known from NB was found in a sedge (*Carex*) marsh and was collected by treading a sphagnum and *Carex* hummock into water during June.

##### Stenus (Stenus) vicinus

Taxon classificationAnimaliaColeopteraStaphylinidae

Casey, 1884

###### Material examined.

**New Brunswick, Restigouche Co.**, Jacquet River Gorge P.N.A. 47.8200°N, 66.0015°W, 13.V.2010, R. P. Webster // Under alders, in leaf litter & moss near small brook in *Carex* marsh (1 ♀, RWC).

###### Distribution in Canada and Alaska.

ON, **NB** (Bousquet et al. 2013).

###### Natural history.

Only one specimen is known from NB. It was sifted from leaf litter and moss under alders near a small brook flowing through a *Carex* marsh. The specimen was collected during May.

##### Stenus (Tesnus) formicetorum

Taxon classificationAnimaliaColeopteraStaphylinidae

Mannerheim, 1843

###### Material examined.

**New Brunswick, Albert Co.**, Caledonia Gorge P.N.A., 45.7930°N, 64.7764°W, 1.VII.2011, R.P. Webster // Small rocky clear cold river margin (Caledonia Creek), sifting drift material (tree bud material) near eddy area (1 ♂, RWC). **Restigouche Co.**, Wild Goose Lake, 420 m elev., 47.8540°N, 68.3219°W, 7.VI.2011, R.P. Webster // Lake margin with emergent *Carex* and grasses, treading *Carex* and grasses (3 ♂, 5 ♀, RWC). **York Co.**, Douglas, Currie Mountain, 45.9844°N, 66.7592°W, 27.V-10.VI.2013, C. Alderson & V. Webster // Mixed forest with *Quercus rubra*, Lindgren funnel trap in canopy of *Quercus rubra* (1 sex undetermined, AFC); Charters Settlement, 45.8456°N, 66.7267°W, 1.V.2010, R.P. Webster // Margin of beaver pond in leaf litter (1 ♂, RWC). **Ontario**, Manitouwadge, Black River, 18.IX.1989, under weeds in dried-up pool, T. Bakker (1 ♂, CNC).

###### Distribution in Canada and Alaska.

YK, AB, MB, **ON**, QC, **NB** (Bousquet et al. 2013). This species is newly recorded from ON and NB.

###### Natural history.

Most specimens of *Stenus formicetorum* were found along a lake margin with emergent vegetation of *Carex* and grasses. Adults were collected by treading vegetation into water. One individual was sifted from leaf litter along the margin of a beaver pond, one was sifted from drift material (tree bud material) near an eddy area along a small rocky, clear, cold river margin, and another adult was captured in a Lindgren funnel trap in the canopy of a red oak in a mixed forest. This species was collected in May, June, and July in NB, and September in ON.

#### Subfamily Euaesthetinae C.G. Thomson, 1859

This is a small subfamily, with 28 species reported from North America by Newton et al. (2000). Sixteen species in three genera were reported from Canada by Bousquet et al. (2013), including four species from NB. Puthz (2014), in a review of North American species of *Euaesthetus*, lists 15 species for Canada and 13 for NB, nine of which were newly recorded for the province. *Euaesthetus chantali* Puthz, *Euaesthetus iripennis* Casey, *Euaesthetus laeviusculus* Mannerheim, *Euaesthetus ganglbauri* Bernhauer, and *Euaesthetus mundulus* Casey were new provincial records; *Euaesthetus floridae* Casey was a new Canadian record. The following species were newly described from specimens, in part from NB: *Euaesthetus blanchardi* Puthz, *Euaesthetus hermani* Puthz, and *Euaesthetus websteri* Puthz (Puthz 2014). Puthz (2014) noted that the holotype of *Euaesthetus websteri* was in the Reginald Webster collection (RWC). The holotype has now been deposited in the CNC. Most species of this subfamily in Canada occur in the genus *Euaesthetus* (Bousquet et al. 2013, Puthz 2014).

Members of this subfamily that occur in Canada occur in *Salix* litter along mountain streams (*Nordenskioldia*), forest litter (*Stictocranius*), forest litter and moss in wetland habitats, on muddy soil near wetlands (*Euaesthetus*), and log-leaf litter, tree holes, forest litter, and decaying organic material such as compost (*Edaphus*) (Puthz 1974, 2010, 2014).

Here, we report *Edaphus lederi* Eppelsheim, which is a new species and genus for Canada and NB.

##### Tribe Euaesthetini C.G. Thomson, 1859

###### Edaphus lederi

Taxon classificationAnimaliaColeopteraStaphylinidae

Eppelsheim, 1878†

Fig. 42

####### Material examined.

**Canada, New Brunswick, York Co.**, Charters Settlement, 45.8395°N, 66.7391°W, 5.IX.2009, R.P. Webster, coll. // Mixed forest, in pile of decaying corncobs and cornhusks (1, RWC); same locality and habitat data but 3.IX.2010 (4, RWC).

####### Distribution in Canada and Alaska.

**NB (new Canadian record).** Puthz (2010) synonymized *Edaphus beszedesi* Reitter (Type locality Lincoln, Nebraska (Reitter 1914)) with *Edaphus lederi*, so all previous records of *Edaphus beszedesi* are *Edaphus lederi*. Puthz considered this species to be Palaearctic and adventive to North America and reported it from IL and KS in the USA. There is an additional specimen in CNC determined by Puthz from AL, indicating that this species is widespread in the USA. This species is widespread in central and southern Europe and may have been introduced into North America with leaf litter or other vegetable debris (Puthz 1974). Puthz (2010) reported it from Taiwan, where it is adventive. This is the first record of this genus for Canada.

####### Natural history.

The five specimens of this species from NB were sifted from a pile of decaying, moldy corncobs and cornhusks in September. It was reported from a corncob pile in IL and decaying vegetation in KS (Puthz 1974).

**Figure 42.**
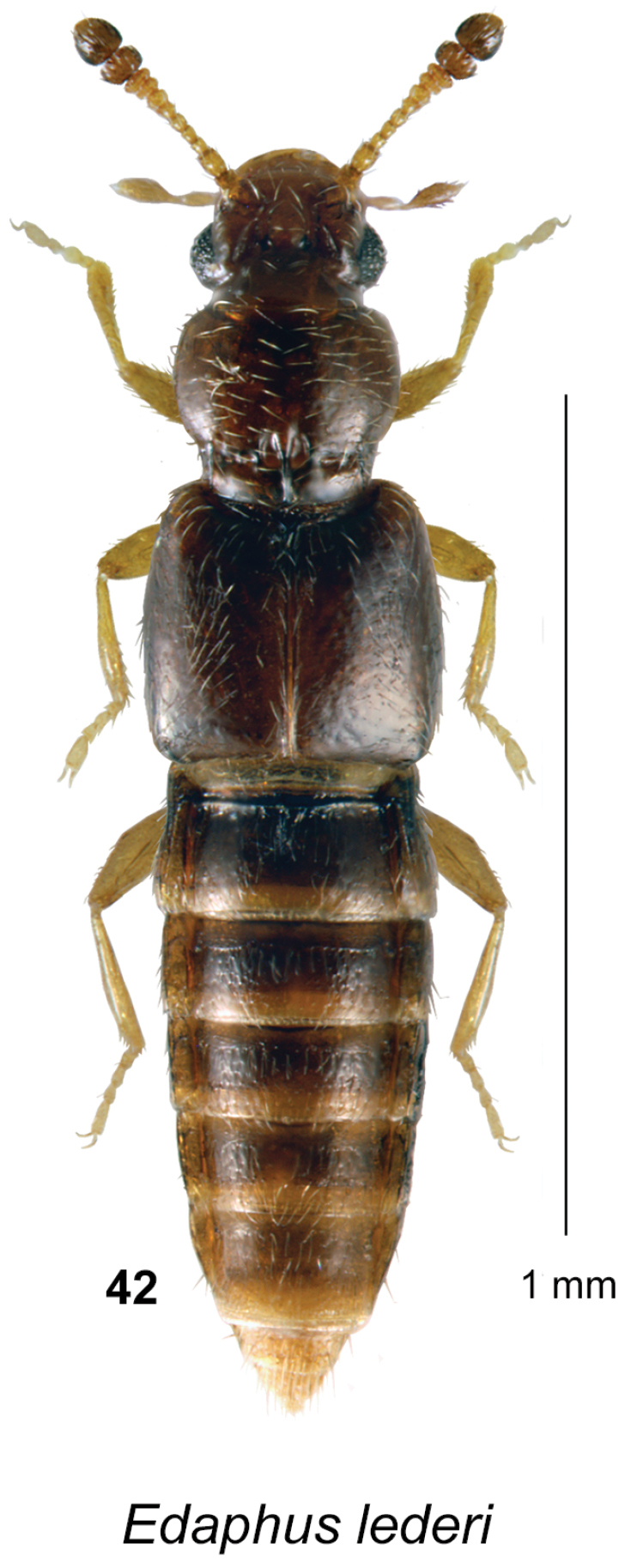
*Edaphus lederi* Eppelsheim: habitus in dorsal view.

#### Subfamily Pseudopsinae Ganglbauer, 1895

##### Pseudopsis sagitta

Taxon classificationAnimaliaColeopteraStaphylinidae

Herman, 1975

###### Material examined.

**New Brunswick, Restigouche Co.**, Dionne Brook P.N.A., 47.9064°N, 68.3441°W, 31.V-15.VI.2011, 15-27.VI.2011, M. Roy & V. Webster // Old-growth white spruce & balsam fir forest, flight intercept traps (3 ♂, RWC).

###### Distribution in Canada and Alaska.

AK, BC, AB, MB, ON, QC, **NB** (Bousquet et al. 2013).

###### Natural history.

The three NB specimens of this boreal species were captured in flight intercept traps in June in an old-growth white spruce (*Picea glauca* (Moench) Voss) and balsam fir forest in the extreme northwestern part of the province.

#### Subfamily Paederinae Fleming, 1821

##### Tribe Paederini Fleming, 1821

###### Subtribe Astenina Hatch, 1957

####### Astenus

Taxon classificationAnimaliaColeopteraStaphylinidae

Dejean, 1833

######## Note.

Newton et al. (2000) reported 24 species of *Astenus* from North America; seven species are reported from Canada and two (*Astenus cinctus* (Say) and *Astenus discopunctatus* (Say) from NB (Bousquet et al. 2013). Here, we report two additional species from the province.

Downie and Arnett (1996) provided a key to the species of northeastern North America which was used to identify the specimens reported below. The species occurring in New Brunswick and eastern Canada have good external and male genitalic (shape of aedeagus) characters for separating species. However, since there have been no revisions of this genus since Casey’s (1905) key, the species names used below should be treated as provisional.

####### Astenus americanus

Taxon classificationAnimaliaColeopteraStaphylinidae

(Casey, 1905)

######## Material examined.

**New Brunswick, Charlotte Co.**, 5.2 km NW of Pomeroy Ridge, 45.3087°N, 67.4362°W, 5.VI.2008, R.P. Webster // Red maple swamp, in leaf litter and in near vernal pool (1 ♂, RWC). **Northumberland Co.**, 12 km SSE of Upper Napan [Goodfellow Brook P.N.A.], 46.8943°N, 65.3796°W, 7.VI.2006, R..P. Webster // Eastern white cedar swamp, in moss & leaf litter (1 ♂, RWC). **Saint John Co.**, Chance Harbour off Rt. 790, 45.1355°N, 66.3672°W, 15.V.2006, R.P. Webster // Calcareous fen, in sphagnum & litter in depressions with *Carex* (1 ♂, RWC). **York Co.**, Charters Settlement, 45.8428°N, 66.7279°W, 15.IV.2005, R.P. Webster // Mixed forest, small sedge marsh in moist grass litter (1 sex undetermined, RWC); same locality and collector but 45.8267°N, 66.7343°W, 16.IV.2005 // *Carex* marsh, in litter & sphagnum at base of tree (2 sex undetermined, RWC); New Maryland, off Hwy 2, E of Baker Brook, 45.8760°N, 66.6252°W, 6.IV.2005, 26.IV.2005, R.P. Webster // Old-growth cedar swamp, in moss & litter at base of cedar (1 ♂, 1 sex undetermined, RWC); Canterbury, trail to Browns Mtn. Fen, 45.9033°N, 67.6260°W, 2.V.2005, M. Giguère & R. Webster // Mixed forest with cedar, margin of vernal pond in moist leaf litter (1 sex undetermined, RWC); Rt. 645 at Beaver Brook, 45.6840°N, 66.8679°W, 3.V.2008, R.P. Webster // Red maple/alder swamp, and in moist leaves near small vernal pool near small stream (1 ♂, RWC).

######## Distribution in Canada and Alaska.

ON, QC, **NB** (Bousquet et al. 2013).

######## Natural history.

*Astenus americanus* was found in moist leaf litter, sphagnum and leaf litter, and in moist leaves on the margin of a vernal pond in forested wetlands. These included a red maple swamp, eastern white cedar swamps, mixed forests with cedar, a red maple/alder swamp, and a small sedge marsh in a mixed forest. Some individuals were found in a calcareous fen and a *Carex* marsh. Adults were collected in April, May, and June.

####### Astenus brevipennis

Taxon classificationAnimaliaColeopteraStaphylinidae

(Austin, 1877)

######## Material examined.

**New Brunswick, Northumberland Co.**, 12 km SSE of Upper Napan [Goodfellow Brook P.N.A.], 46.8943°N, 65.3796°W, 7.VI.2006, R.P. Webster // Eastern white cedar swamp, in moss & leaf litter (2 ♂, 2 sex undetermined, RWC); same locality data and collector but 23.V.2007 // Old-growth, wet eastern white cedar swamp, in litter, grasses & moss on hummocks near water [pools] (1 ♂, 2 sex undetermined, RWC).

######## Distribution in Canada and Alaska.

MB, ON, **NB** (Bousquet et al. 2013).

######## Natural history.

This species was sifted from moss and leaf litter, and litter, grasses, and moss on hummocks near water in an old-growth eastern white cedar swamp. Adults were found in May and June.

###### Subtribe Medonina Casey, 1905

####### Medon (Medon) fusculus

Taxon classificationAnimaliaColeopteraStaphylinidae

(Mannerheim, 1830)†

######## Material examined.

**New Brunswick, York Co.**, Fredericton, Odell Park, 45.9570°N, 66.6695°W, 7.IX.2005, R.P. Webster // Mixed forest, in compost (decaying plant material) (1 ♂, RWC). **Ontario**, Milldale, 45°56'08N 80°35'08W, 25.V.2011, A. Davies, beech and poplar litter in deep ravine (8, CNC). **Quebec**, Johnville, La Framboisière de l’Estrie, 24.V.1989, C. Lévesque (1, CNC); Compton, 2.VI.2014 (1 ♀), 9.VI.2014 (1 ♂), 16.VI.2014 (1 ♀), 23.VI.2014 (1 ♂), C. Lévesque, pièges à fosse, en bordure d’un verger, (all coll. C. Lévesque).

######## Distribution in Canada and Alaska.

ON, QC, **NB** (Bousquet et al. 2013). The Palaearctic *Medon fusculus* is adventive to North America and was first reported from QC in the checklist by Campbell and Davies (1991). Brunke and Marshall (2011) provided the first documented records for North America. Here, we present the first record from NB, as well as the data on which the distribution given in Bousquet et al. (2013) was based (CNC).

######## Natural history.

In the Palaearctic, *Medon fusculus* occurs in leaf litter and compost (Assing 2004). The sole specimen from NB was found in a compost pile in a mixed forest. Specimens from ON were sifted from deciduous litter in a small fragment of mature forest, collected from under a rock, in pitfall traps and canopy traps along hedgerows (Brunke and Marshall 2011), and sifted from damp beech and poplar litter by a stream in a deep ravine on agricultural land (CNC). The QC specimens were collected in pitfall traps on a raspberry plantation and at the edge of an orchard growing apples, pears, and plums.

###### Subtribe Scopaeina Mulsant & Rey, 1878

####### Orus (Pycnorus) dentiger

Taxon classificationAnimaliaColeopteraStaphylinidae

(LeConte, 1880)

######## Material examined.

**New Brunswick, Saint John Co.**, Chance Harbour, 45.1156°N, 66.3610°W, 7.V.2006, R.P. Webster // In decaying seaweed on gravel beach (1 ♂, RWC); Chance Harbour, off Cranberry Head Rd., 45.1357°N, 66.3451°W, 12.V.2008, R.P. Webster // Barrier beach, in decaying sea wrack on gravel & sand (1 ♀, RWC).

######## Distribution in Canada and Alaska.

AK, AB, MB, ON, QC, **NB** (Bousquet et al. 2013).

######## Natural history.

In NB, two specimens of *Orus dentiger* were sifted from decaying sea wrack on gravel sea beaches during May. Elsewhere, this species has been collected from March to November under stones, in soil samples, on lake shores, in sphagnum moss on the margin of a tamarack (*Larix laricina* (Du Roi) Koch) marsh (Blatchley 1910, Herman 1965), from clumps of moss and grass in a swamp, and under a log on a riverbank (CNC).

####### Scopaeus (Scopaeus) minutus

Taxon classificationAnimaliaColeopteraStaphylinidae

Erichson, 1840†

######## Material examined.

**New Brunswick, York Co.**, Fredericton, at St. John River, 45.9588°N, 66.6254°W, 7.VI.2005, R.P. Webster // River margin, in flood debris (1 ♀, RWC); Charters Settlement, 45.8395°N, 66.7391°W, 30.IV.2005, 5.VI.2007, 20.IX.2007, 30.VI.2008, R.P. Webster // Residential lawn, on soil at base of lawn grass (2 ♂, 7 sex undetermined, RWC).

######## Distribution in Canada and Alaska.

ON, QC, **NB** (Bousquet et al. 2013). This adventive species from the Palaearctic was first reported in North America from Montreal, QC by Frisch et al. (2002), followed by additional records from ON reported by Brunke and Marshall (2011).

######## Natural history.

In the Palaearctic, *Scopaeus minutus* is usually found in early successional habitats (Boháč 1985) and drier habitats than other members of this genus (Frisch et al. 2002). Specimens from ON were caught in passive traps in soybean fields and woodlot edges (Brunke and Marshall 2011). Most NB specimens were found on soil at the base of grass in a residential lawn. One individual was sifted from flood debris along a river margin.

###### Subtribe Stilicina Casey, 1905

####### Rugilis ceylanensis

Taxon classificationAnimaliaColeopteraStaphylinidae

(Kraatz, 1859)†

######## Material examined.

**New Brunswick, York Co.**, Charters Settlement, 45.8395°N, 66.7391°W, 20.VIII.2006, 22.VIII.2006, 26.IX.2007, 23.IX.2009, 1.X.2009, R.P. Webster // Mixed forest, in decaying (moldy) corncobs & cornhusks (2 ♂, 8 sex undetermined, RWC).

######## Distribution in Canada and Alaska.

ON, QC, **NB** (Bousquet et al. 2013). *Rugilus ceylanensis* occurs in the southern and eastern Palaearctic and Oriental regions, New Guinea, and Hawaii where it is adventive (Hoebeke 2010, Assing 2012). Hoebeke (2010) reported this adventive species for the first time for North America from several states in the USA, and ON and QC in Canada.

######## Natural history.

All specimens of *Rugilis ceylanensis* from NB were collected from a pile of decaying moldy corncobs and cornhusks. Elsewhere in the USA and Canada, this species was found in leaf piles, rotten leaves and logs, detritus, horse dung, and carrion (Hoebeke 2010), and at the edge of an orchard growing apples, pears, and plums (coll. C. Lévesque). In Europe, adults were found in compost heaps, mammal dung, carrion, and along lakeshores and riverbanks (Assing 2012).

#### Subfamily Staphylininae Latreille, 1802

##### Tribe Staphylinini Latreille, 1802

###### Subtribe Philonthina Kirby, 1837

####### Bisnius fimetarius

Taxon classificationAnimaliaColeopteraStaphylinidae

(Gravenhorst, 1802)†

######## Material examined.

**New Brunswick, Restigouche Co.**, Dionne Brook P.N.A., 47.9030°N, 68.3503°W, 25.V.2011, R.P. Webster // Old-growth northern hardwood forest, in moose dung (1 ♂, 1 ♀, RWC).

######## Distribution in Canada and Alaska.

QC, **NB**, NF (Bousquet et al. 2013). *Bisnius fimetarius* is a Palaearctic species (Smetana 2004) previously known to be adventive to North America in NF and QC (Smetana 1965, 1995).

######## Natural history.

Smetana (1995) reported this species in various kinds of organic material such as dung and carrion, usually near human settlements. The two specimens from NB were found in moose dung in an old-growth northern hardwood forest.

####### Bisnius pugetensis

Taxon classificationAnimaliaColeopteraStaphylinidae

(Hatch, 1957)

######## Material examined.

**New Brunswick, York Co.**, Keswick Ridge, 45.9962°N, 66.8781°W, 25.V.2015, R.P. Webster // Margin field/hardwood forest, in litter in entrance to *Marmota monax* burrow (1 ♀, RWC).

######## Distribution in Canada and Alaska.

BC, AB, SK, MB, ON, QC, **NB** (Bousquet et al. 2013).

######## Natural history.

Smetana (1995) reported this species from burrows of various mammals such as gophers, *Thomomys*, *Marmota*, and fox. The NB specimen was collected from litter in the entrance of a *Marmota monax* (L.) (groundhog) burrow.

####### Gabrius lysippus

Taxon classificationAnimaliaColeopteraStaphylinidae

Smetana, 1995

######## Material examined.

**New Brunswick, Albert Co.**, Caledonia Gorge P.N.A., 45.7930°N, 64.7764°W, 1.VII.2011, R. P. Webster //Small rocky clear-cold river (Caledonia Creek), splashing exposed rocks covered with moss in middle of river (2 ♀, NBM; 3 ♂, 1 ♀, RWC); Caledonia Gorge P.N.A., 45.7686°N, 64.8065°W, 2.VII.2011, R.P. Webster // McKinley Brook, rocky cool water, shaded brook, in moss on large rocks (2 ♂, 1 ♀, RWC); Caledonia Gorge P.N.A., 45.8432°N, 64.8411°W, 5.VII.2011, R.P. Webster // Turtle Creek, rocky, cold water & shaded creek, in saturated moss on rocks (1 ♂, NBM); Caledonia Gorge P.N.A., 45.7935°N, 64.7744°W, 22.V.2012, R.P. Webster // Crooked Creek, cold clear rocky stream, in *Carex* hummock in stream (1 ♀, RWC). **Queens Co.**, C.F.B. Gagetown, 45.7516°N, 66.1866°W, 9-22.V.2013, C. Alderson & V. Webster // Old mixed forest with *Quercus rubra*, Lindgren funnel trap in canopy of *Quercus rubra* (1 ♂, AFC).

######## Distribution in Canada and Alaska.

QC, **NB** (Bousquet et al. 2013).

######## Natural history.

Smetana (1995) reported *Gabrius lysippus* from wet moss on rocks at streams or along the margin of streams. This species was found in similar habitats in NB. Adults were collected by splashing exposed rocks covered with moss in the middle of a small rocky, cold river, and from moss and saturated moss on rocks in shaded brooks. One individual was found in a *Carex* hummock in a stream and one was caught in a Lindgren funnel trap in the canopy of a red oak. Small streams with moss-covered rocks were present at the latter site. Adults were collected in May and July.

## Supplementary Material

XML Treatment for Olophrum boreale

XML Treatment for Phyllodrepa humerosa

XML Treatment for Proteinus acadiensis

XML Treatment for Proteinus hughesi

XML Treatment for Proteinus parvulus

XML Treatment for Proteinus pseudothomasi

XML Treatment for Proteinus sweeneyi

XML Treatment for Mycetoporus rufohumeralis

XML Treatment for Sepedophilus immaculatus

XML Treatment for Tachinus (Tachinus) elongatus

XML Treatment for Bledius basalis

XML Treatment for Bledius opaculus

XML Treatment for Carpelimus

XML Treatment for Carpelimus difficilis

XML Treatment for Carpelimus erichsoni

XML Treatment for Carpelimus gracilis

XML Treatment for Carpelimus lacustris

XML Treatment for Carpelimus mundus

XML Treatment for Carpelimus obesus

XML Treatment for Carpelimus probus

XML Treatment for Carpelimus pusillus

XML Treatment for Carpelimus quadripunctatus

XML Treatment for Carpelimus rivularis

XML Treatment for Carpelimus spretus

XML Treatment for Carpelimus subtilis

XML Treatment for Carpelimus weissi

XML Treatment for Ochthephilus planus

XML Treatment for Platystethus (Craetopycrus) degener

XML Treatment for Thinodromus corvinus

XML Treatment for Brachycepsis subpunctata

XML Treatment for Dianous nitidulus

XML Treatment for Stenus (Hemistenus) sibiricus

XML Treatment for Stenus (Hypostenus) alexanderi

XML Treatment for Stenus (Hypostenus) destitutus

XML Treatment for Stenus (Hypostenus) punctatus

XML Treatment for Stenus (Stenus) carinicollis

XML Treatment for Stenus (Stenus) comma comma

XML Treatment for Stenus (Stenus) difficilis

XML Treatment for Stenus (Stenus) egenulus

XML Treatment for Stenus (Stenus) fulvoguttatus

XML Treatment for Stenus (Stenus) pluto

XML Treatment for Stenus (Stenus) pumilio

XML Treatment for Stenus (Stenus) vicinus

XML Treatment for Stenus (Tesnus) formicetorum

XML Treatment for Edaphus lederi

XML Treatment for Pseudopsis sagitta

XML Treatment for Astenus

XML Treatment for Astenus americanus

XML Treatment for Astenus brevipennis

XML Treatment for Medon (Medon) fusculus

XML Treatment for Orus (Pycnorus) dentiger

XML Treatment for Scopaeus (Scopaeus) minutus

XML Treatment for Rugilis ceylanensis

XML Treatment for Bisnius fimetarius

XML Treatment for Bisnius pugetensis

XML Treatment for Gabrius lysippus

## References

[B1] Assing V (2004) A revision of the *Medon* species of the eastern Mediterranean and adjacent regions. Bonner Zoologische Beiträge 52: 33–82.

[B2] Assing V (2012) The *Rugilus* species of the Palaearctic and Oriental regions (Coleoptera: Staphylinidae: Paederinae). Stuttgarter Beiträge zur Naturkunde A, Neue Serie 5: 115–190.

[B3] Bernhauer M Schubert K (1911) Staphylinidae II. (Pars 29) In: Junk W Schenkling S (Eds) Coleopterorum Catalogus. Volumen 5. Staphylinidae. Junk, Berlin, 87–190.

[B4] Betz O (1996) Function and evolution of the adhesion-capture apparatus of *Stenus* species (Coleoptera, Staphylinidae). Zoomorphology 116: 15–34. doi: 10.1007/BF02526926

[B5] Betz O (1998) Comparative studies on the predatory behaviour of *Stenus* spp. (Coleoptera: Staphylinidae): the significance of its specialized labial apparatus. Journal of Zoology (London) 244: 527–544. doi: 10.1111/j.1469-7998.1998.tb00058.x

[B6] Betz O (1999) A behavioural inventory of adult *Stenus* species (Coleoptera: Staphylinidae). Journal of Natural History 33: 1691–1712. doi: 10.1080/002229399299806

[B7] Blatchley WS (1910) An illustrated descriptive catalogue of the Coleoptera or beetles (exclusive of the Rhynchophora) known to occur in Indiana. The Nature Publishing Co., Indianapolis, Indiana, 1386 pp.

[B8] Boháč J (1985) Review of the subfamily Paederinae (Coleoptera, Staphylinidae) in Czechoslovakia. Acta Entomologica Bohemoslavaca 82: 360–385.

[B9] Bouchard P Bousquet Y Davies AE Alonso-Zarazaga MA Lawrence JF Lyal CHC Newton AF Ried CAM Schmitt M Ślipiński SA Smith ABT (2011) Family-group names in Coleoptera (Insecta). ZooKeys 88: 1–972. doi: 10.3897/zookeys.88.807 21594053 10.3897/zookeys.88.807 PMC3088472

[B10] Bousquet Y Bouchard P Davies AE Sikes D (2013) Checklist of beetles (Coleoptera) of Canada and Alaska. Second edition Pensoft Series Faunistica No. 109, Sofia-Moscow, 402 pp. 10.3897/zookeys.360.4742 PMC3867111 24363590

[B11] Brunke AJ Marshall SA (2011) Contributions to the faunistics and bionomics of Staphylinidae (Coleoptera) in northeastern North America: discoveries made through study of the University of Guelph Insect Collection, Ontario, Canada. ZooKeys 75: 29–68. doi: 10.3897/zookeys/75.767 21594139 10.3897/zookeys.75.767 PMC3088045

[B12] Callot H (2013) Carpelimus (Trogophloeus) erichsoni (Sharp, 1871), nouvelle espèce pour la faune de France (Coleoptera Staphylinidae Oxytelinae). Entomologiste (Paris) 69: 315–316.

[B13] Campbell JM (1973) A revision of the genus *Tachinus* (Coleoptera: Staphylinidae) of North and Central America. Memoirs of the Entomological Society of Canada 90: 1–137. doi: 10.4039/entm10590fv

[B14] Campbell JM (1983) A revision of the North American Omaliinae (Coleoptera: Staphylinidae). The genus *Olophrum* Erichson. The Canadian Entomologist 115: 577–622. doi: 10.4039/Ent115577-6

[B15] Campbell JM (1991) A revision of the genera *Mycetoporus* Mannerheim and *Ishnosoma* Stephens (Coleoptera : Staphylinidae : Tachyporinae) of North and Central America. Memoirs of the Entomological Society of Canada No. 156: 1–169. doi: 10.4039/entm1123156fv

[B16] Campbell JM Davies A (1991) Family Staphylinidae. In: Bousquet Y (Ed.) Checklist of beetles of Canada and Alaska. Research Branch, Agriculture Canada, Ottawa, Publication 1861/E, 86–124.

[B17] Casey TL (1889) A preliminary monograph of the North American species of *Trogophloeus*. Annals of the New York Academy of Sciences 4(10/11): 322–383. doi: 10.1111/j.1749-6632.1889.tb57043.x

[B18] Casey TL (1905) A revision of the American Paederini. Transactions of the Academy of Science of St. Louis 15: 17–248.

[B19] Downie NM Arnett RH Jr (1996) The Beetles of Northeastern North America, Volumes 1 and 2. Sandhill Crane Press, Gainesville, Florida, 1721 pp.

[B20] Fauvel A (1871) Faune Gallo-Rhénane ou descriptions des insectes qui habitent la France, la Belgique, la Hollande, le Luxembourg, les provinces Rhénanes et la Valais avec tableaux synoptiques et planches gravées. Bulletin de la Société Linnéenne de Normandie 5([1869–70]): 27–192.

[B21] Fauvel A (1889) Liste des coléoptères communs à l’Europe et à l’Amérique du Nord. D’après le catalogue de M. J. Hamilton. Avec remarques et additions. Revue d’Entomologie 8: 92–174.

[B22] Frank JH (1982) The parasites of the Staphylinidae (Coleoptera): a contribution towards an encyclopedia of the Staphylinidae. Bulletin / University of Florida Agricultural Experiment Station No. 824: vii + 118 pp.

[B23] Frisch J Burckhardt D Wolters V (2002) Rove beetles of the subtribe Scopaeina Mulsant and Rey (Coleoptera: Staphylinidae) in the West Palaearctic: phylogeny, biogeography and species catalogue. Organisms Diversity & Evolution 2: 27–53. doi: 10.1078/1439-6092-00032

[B24] Gildenkov MY (2015) Fauna *Carpelimus* Starogo Sveta (Coleoptera: Staphylinidae). Fauna *Carpelimus* of the Old World. Monografiya. Smolensk, Izdatel’stvo SmolGU, 413 pp.

[B25] Hatch MH (1957) The beetles of the Pacific Northwest. Part II: Staphyliniformia. University of Washington Press, Seattle, ix + 384 pp.

[B26] Herman LH Jr (1965) Revision of *Orus*. II Subgenera *Orus*, *Pycnorus* and *Nivorus* (Coleoptera: Staphylindae). The Coleopterists’ Bulletin 19: 73–90.

[B27] Herman LH Jr (1976) Revision of *Bledius* and related genera, Part II. The *armatus*, *basalis*, and *melanocephalus* groups (Coleoptera, Staphylinidae, Oxytelinae). Bulletin of the American Museum of Natural History 157: 71–172.

[B28] Hoebeke ER (2010) *Rugilus ceylanensis* (Kraatz) (Coleoptera: Staphylinidae: Paederinae): a south Asian rove beetle new to North America. Proceedings of the Entomological Society of Washington 112: 508–516. doi: 10.4289/0013-8797.112.4.508

[B29] Hubbard HG Schwarz EA (1878) The Coleoptera of Michigan. [includes:] List of Coleoptera found in the Lake Superior region [pp. 627–643]; Contribution to a list of the Coleoptera of the lower peninsula of Michigan [pp. 643–666]. Proceedings of the American Philosophical Society 17 (No. 101): 593–666.

[B30] Hughes CC Johns RC Sweeney JD (2014) A technical guide to installing beetle traps in the upper crown of trees. Journal of the Acadian Entomological Society 10: 12–18.

[B31] Jenkins MF (1960) On the method by which *Stenus* and *Dianous* (Coleoptera: Staphylinidae) return to the banks of a pool. Transactions of the Royal Entomological Society of London 112: 1–14. doi: 10.1111/j.1365-2311.1960.tb00487.x

[B32] Klimaszewski J Sweeney J Price J Pelletier G (2005) Rove beetles (Coleoptera: Staphylinidae) in red spruce stands, eastern Canada: diversity, abundance, and descriptions of new species. The Canadian Entomologist 137: 1–48. doi: 10.4039/n03-123

[B33] Klimaszewski J Langor D Savard K Pelletier G Chandler DS Sweeney J (2007) Rove beetles (Coleoptera: Staphylinidae) in yellow birch-dominated stands of southeastern Quebec, Canada: Diversity, abundance, and description of a new species. The Canadian Entomologist 139: 793–833. doi: 10.4039/n06-057

[B34] Klimaszewski J Webster RP Langor DW Bourdon C Jacobs J (2013) Review of Canadian species of the genus *Dinaraea* Thomson, with descriptions of six new species (Coleoptera, Staphylinidae, Aleocharinae, Athetini). ZooKeys 327: 65–101. doi: 10.3897/zookeys.327.5908 24167422 10.3897/zookeys.327.5908 PMC3804766

[B35] Klimaszewski J Webster RP Langor DW Bourdon C Hammond HEJ Pohl GR Godin B (2014) Review of Canadian species of the genera *Gnathusa* Fenyes, *Mniusa* Mulsant & Rey and *Ocyusa* Kraatz (Coleoptera, Staphylinidae, Aleocharinae). ZooKeys 412: 9–40. doi: 10.3897/zookeys.412.7282 24899860 10.3897/zookeys.412.7282 PMC4042694

[B36] Klimaszewski J Webster RP Bourdon C Pelletier G Godin B Langor DW (2015a) Review of Canadian species of the genus *Mocyta* Mulsant & Rey (Coleoptera, Staphylinidae, Aleocharinae), with the description of a new species and a new synonymy. ZooKeys 487: 111–139. doi: 10.3897/zookeys.487.9151 25829852 10.3897/zookeys.487.9151 PMC4366688

[B37] Klimaszewski J Webster RP Sikes D Bourdon C Labrecque M (2015b) A review of Canadian and Alaskan species of the genera *Clusiota* Casey and Atheta Thomson, subgenus Microdota Mulsant & Rey (Coleoptera, Staphylinidae, Aleocharinae). ZooKeys 524: 103–136. doi: 10.3897/zookeys.524.6105 26478708 10.3897/zookeys.524.6105 PMC4602293

[B38] Klimaszewski J Webster RP Langor DW Sikes D Godin B Bourdon C Ernst C (2016) A review of Canadian and Alaskan species of the genus *Liogluta* Thomson, and descriptions of three new species (Coleoptera, Staphylinidae). In: Webster RP Bouchard P Klimaszewski J (Eds) The Coleoptera of New Brunswick and Canada: providing baseline biodiversity and natural history data. ZooKeys 573: 217–256. doi: 10.3897/zookeys.573.7878 10.3897/zookeys.573.7878 PMC4829928 27110169

[B39] LeConte JL (1877) On certain genera of Staphylinidae Oxytelini, Piestidae, and Micropeplidae, as represented in the fauna of the United States. Transactions of the American Entomological Society 6: 213–252. doi: 10.2307/25076322

[B40] Lindgren BS (1983) A multiple funnel trap for scolytid beetles (Coleoptera). The Canadian Entomologist 115: 299–302. doi: 10.4039/Ent115299-3

[B41] Majka CG Klimaszewski J Lauff RF (2008) The coastal rove beetles (Coleoptera, Staphylinidae) of Atlantic Canada: a survey and new records. ZooKeys 2: 115–150. doi: 10.3897/zookeys.2.2

[B42] Makranczy Gy (2002) The fauna of the Fertő-Hanság National Park Volume 1. Mahunka S (Ed.) (Natural history of the national parks of Hungary, 12) Hungarian Natural History Museum, Budapest, 417–421.

[B43] Makranczy Gy (2014) Revision of the genus *Ochthephilus* Mulsant & Rey, 1856 (Coleoptera: Staphylinidae, Oxytelinae). Revue Suisse de Zoologie 121: 457–694.

[B44] Newton AF Thayer MK Ashe JS Chandler DS (2000) [2001] Family 22. Staphylinidae Latreille, 1802. In: Arnett RH Thomas MC (Eds) American Beetles. Volume 1. Archostemata, Myxophaga, Adephaga, Polyphaga: Staphyliniformia. CRC Press, Boca Raton, Florida, 272–418.

[B45] Notman H (1920) Coleoptera collected at Windsor, Broome Co., N. Y., 26 May to 5 June, 1918, with notes and descriptions. Journal of the New York Entomological Society 28(2): 178–194.

[B46] Notman H (1924) Two new staphylinids from Cranberry Lake, New York. Technical Publication No. 17, NY State College of Forestry, Syracuse University XXIV(22): 270–272.

[B47] O’Keefe ST (2000) [2001] Family 20. Scydmaenidae Leach, 1815. In: Arnett RH Thomas MC (Eds) American Beetles. Volume 1. Archostemata, Myxophaga, Adephaga, Polyphaga: Staphyliniformia. CRC Press, Boca Raton, Florida, 259–267.

[B48] Puthz V (1974) A new revision of the Nearctic *Edaphus* -species and remarks on other North American Euaesthetinae (Coleoptera, Staphylinidae). Revue Suisse de Zoologie 81: 911–932. doi: 10.5962/bhl.part.76051

[B49] Puthz V (2010) *Edaphus* aus Taiwan (Coleoptera: Staphylinidae) 101. Beitrag zur Kenntnis der Euaesthetinen. Revue Suisse de Zoologie 116: 265–336. doi: 10.5962/bhl.part.117785

[B50] Puthz V (2014) Nordamerikanische Arten der Gattung *Euaesthetus* Gravenhorst (Coleoptera, Staphylinidae) 115. Beitrag zur Kenntnis der Euaesthetinen. Linzer Biologische Beiträge 46: 845–876.

[B51] Reitter E (1914) Übersicht der bekannten Arten der Coleopteren-Gattung *Edaphus* Leconte (Staphyl.) aus Europa und den angrenzenden Ländern. Berliner Entomologische Zeitschrift 58 [1913]: 188–189.

[B52] Scheerpeltz O (1933) Staphylinidae VII (Pars 129). Supplementum I In: Junk W Schenkling S (Eds) Coleopterorum Catalogus. Volumen VI. Staphylinidae. Junk, Berlin, 989–1500.

[B53] Schmid R (1988) Morphologische Anpassungen in einem Räuber-Beute-System: Ameisenkäfer (Scydmaenidae, Staphylinoidea) und gepanzerte Milben (Acari). Zoologische Jahrbücher, Abteilung für Systematik, Ökologie und Geographie der Tiere 115: 207–228.

[B54] Schülke M (2011) Zur identität von *Sepedophilus immaculatus* Stephens (Coleoptera, Staphylinidae, Tachyporinae). Linzer biologische Beiträge 43: 1609–1615.

[B55] Schülke M Smetana A (2015) Staphylinidae. In: Löbl I Löbl D (Eds) Catalogue of Palearctic Coleoptera. Volume 2/1 revised and updated. Hydrophiloidea - Staphylinoidea. Brill, Leiden and Boston, 304–900.

[B56] Schuster R (1966) Über den Beutefang des Ameisenkäfers *Cephennium austriacum* Reiter [sic]. Naturwissenschaften 53: 113. doi: 10.1007/BF00601487

[B57] Sharp D (1876) Contribution to a fauna of the Amazon Valley. Coleoptera-Staphylinidae. Transactions of the Entomological Society of London 1876: 27–424.

[B58] Smetana A (1965) Staphylinini und Quediini (Col., Staphylinidae) von Newfoundland, Südost-Labrador und Nova Scotia (59. Beiträge zur Kenntnis der Staphyliniden). Acta Entomologica Fennica 20: 1–60.

[B59] Smetana A (1995) Rove beetles of the subtribe Philonthina of America north of Mexico (Coleoptera: Staphylinidae). Classification, phylogeny and taxonomic revision. Memoirs on Entomology, International No. 3, x + 946 pp.

[B60] Smetana A (2004) Family Staphylinidae Latrielle, 1802 [subfamilies Piestinae-Staphylininae]. In: Löbl I Smetana A (Eds) Catalogue of Palaearctic Coleoptera. Volume 2. Hydrophiloidea - Histeroidea – Staphylinoidea. Apollo Books, Stenstrup, 504–698.

[B61] Ulke H (1902) A list of the beetles of the District of Columbia. Proceedings of the United States National Museum 25 [1903] (No. 1275): 1–57. doi: 10.5479/si.00963801.1275

[B62] Webster RP Klimaszewski J Pelletier G Savard K (2009) New Staphylinidae (Coleoptera) records with new collection data from New Brunswick, Canada. I. Aleocharinae. In: Majka CG Klimaszewski J (Eds) Biodiversity, biosystematics, and ecology of Canadian Coleoptera II. ZooKeys 22: 171–248. doi: 10.3897/zookeys.22.152

[B63] Webster RP DeMerchant I (2012a) New Staphylinidae (Coleoptera) records with new collection data from New Brunswick, Canada: Oxyporinae. In: Klimaszewski J Anderson R (Eds) Biosystematics and Ecology of Canadian Staphylinidae (Coleoptera) II. ZooKeys 186: 263–271. doi: 10.3897/zookeys.186.2502 22577323 10.3897/zookeys.186.2502 PMC3349197

[B64] Webster RP DeMerchant I (2012b) New Staphylinidae (Coleoptera) records with new collection data from New Brunswick, Canada: Paederinae. In: Klimaszewski J Anderson R (Eds) Biosystematics and Ecology of Canadian Staphylinidae (Coleoptera) II. ZooKeys 186: 273–292. doi: 10.3897/zookeys.186.2504 22577324 10.3897/zookeys.186.2504 PMC3349198

[B65] Webster RP Chandler DS Sweeney JD DeMerchant I (2012a) New Staphylinidae (Coleoptera) records with new collection data from New Brunswick, Canada: Pselaphinae. In: Klimaszewski J Anderson R (Eds) Biosystematics and Ecology of Canadian Staphylinidae (Coleoptera) II. ZooKeys 186: 31–53. doi: 10.3897/zookeys.186.2505 22577317 10.3897/zookeys.186.2505 PMC3349191

[B66] Webster RP Klimaszewski J Sweeney JD DeMerchant I (2012b) New Staphylinidae (Coleoptera) records with new collection data from New Brunswick, and an addition to the fauna of Quebec, Canada: Aleocharinae. In: Klimaszewski J Anderson R (Eds) Biosystematics and Ecology of Canadian Staphylinidae (Coleoptera) II. ZooKeys 186: 83–118. doi: 10.3897/zookeys.186.2655 22577319 10.3897/zookeys.186.2655 PMC3349193

[B67] Webster RP Smetana A Sweeney JD DeMerchant I (2012c) New Staphylinidae (Coleoptera) records with new collection data from New Brunswick and an addition to the fauna of Quebec: Staphylininae. In: Klimaszewski J Anderson R (Eds) Biosystematics and Ecology of Canadian Staphylinidae (Coleoptera) II. ZooKeys 186: 293–348. doi: 10.3897/zookeys.186.2469 22577325 10.3897/zookeys.186.2469 PMC3349199

[B68] Webster RP Sweeney JD DeMerchant I (2012d) New Staphylinidae (Coleoptera) records with new collection data from New Brunswick, Canada: Omaliinae, Micropeplinae, Phloeocharinae, Olisthaerinae, and Habrocerinae. In: Klimaszewski J Anderson R (Eds) Biosystematics and Ecology of Canadian Staphylinidae (Coleoptera) II. ZooKeys 186: 7–29. doi: 10.3897/zookeys.186.2495 22577316 10.3897/zookeys.186.2495 PMC3349190

[B69] Webster RP Sweeney JD DeMerchant I (2012e) New Staphylinidae (Coleoptera) records with new collection data from New Brunswick, Canada: Tachyporinae. In: Klimaszewski J Anderson R (Eds) Biosystematics and Ecology of Canadian Staphylinidae (Coleoptera) II. ZooKeys 186: 55–82. doi: 10.3897/zookeys.186.2491 22577318 10.3897/zookeys.186.2491 PMC3349192

[B70] Webster RP Sweeney JD DeMerchant I (2012f) New Staphylinidae (Coleoptera) records with new collection data from New Brunswick, Canada: Scaphidiinae, Piestinae, Osorinae [sic], and Oxytelinae. In: Klimaszewski J Anderson R (Eds) Biosystematics and Ecology of Canadian Staphylinidae (Coleoptera) II. ZooKeys 186: 239–262. doi: 10.3897/zookeys.186.2506 22577322 10.3897/zookeys.186.2506 PMC3349196

[B71] Webster RP Alderson CA Webster VL Hughes CC Sweeney JD (2016a) Further contributions to the longhorn beetle (Coleoptera, Cerambycidae) fauna of New Brunswick and Nova Scotia, Canada. ZooKeys 552: 109–122. doi: 10.3897/zookeys.552.6039 26865818 10.3897/zookeys.552.6039 PMC4740852

[B72] Webster RP Klimaszewski J Bourdon C Sweeney JD Hughes CC Labrecque M (2016b) Further contributions to the Aleocharinae (Coleoptera, Staphylinidae) fauna of New Brunswick and Canada including descriptions of 27 new species. In: Webster RP Bouchard P Klimaszewski J (Eds) The Coleoptera of New Brunswick and Canada: providing baseline biodiversity and natural history data. ZooKeys 573: 85–216. doi: 10.3897/zookeys.573.7016 10.3897/zookeys.573.7016 PMC4829927 27110168

